# Targeted inhibition of eIF5A^hpu^ suppresses tumor growth and polarization of M2-like tumor-associated macrophages in oral cancer

**DOI:** 10.1038/s41419-023-06109-z

**Published:** 2023-08-31

**Authors:** Jincheng Zeng, Ziyu Ye, Shihong Shi, Yanfang Liang, Qingyu Meng, Qunzhou Zhang, Anh D. Le

**Affiliations:** 1 Department of Oral and Maxillofacial Surgery and Pharmacology, University of Pennsylvania School of Dental Medicine, Philadelphia, PA USA; 2 Dongguan Key Laboratory of Medical Bioactive Molecular Developmental and Translational Research, Guangdong Provincial Key Laboratory of Medical Molecular Diagnostics, Guangdong Medical University, 523808 Dongguan, China; 3 Department of Pathology, Dongguan Hospital Affiliated to Jinan University, Bin-haiwan Central Hospital of Dongguan, 523905 Dongguan, China; 4 Department of Oral & Maxillofacial Surgery, Penn Medicine Hospital of the University of Pennsylvania, Perelman Center for Advanced Medicine, Philadelphia, PA USA

**Keywords:** Targeted therapies, Targeted therapies

## Abstract

Eukaryotic initiation factor 5A2 (eIF5A2) is overexpressed in many types of cancer, and spermidine-mediated eIF5A hypusination (eIF5A^hpu^) appears to be essential to most of eIF5A’s biological functions, including its important role in regulating cancer cell proliferation, epithelial–mesenchymal transition (EMT), and cancer stem cell (CSC) properties as well as immune cell functions. Here we investigated the role of eIF5A^hpu^ in the growth of oral squamous cell carcinoma cells (OSCCs) and OSCC-induced polarization of M2-like tumor-associated macrophages (TAMs). TCGA dataset analysis revealed an overall upregulation in the mRNA expression of *eIF5A2* and several key enzymes involved in polyamine (PA) metabolism in HNSCC, which was confirmed by Western blot and IHC studies. Blocking eIF5A^hpu^ by GC-7 but not the upstream key enzyme activities of PA metabolism, remarkably inhibited cell proliferation and the expression of EMT- and CSC-related genes in OSCC cells. In addition, blocking eIF5A^hpu^ robustly inhibited OSCC-induced M2-like TAM polarization in vitro. More Importantly, blocking eIF5A^hpu^ dramatically retarded tumor growth and infiltration/polarization of M2-like TAM in a syngeneic orthotopic murine tongue SCC model. Thus, eIF5A^hpu^ plays dual functions in regulating tumor cell growth and polarization of M2-TAMs in OSCC.

## Introduction

Head and neck cancer (HNC) is the seventh most common malignancy in the world with about 800,000 new cases diagnosed annually [1]. HNC encompasses a large range of epithelial malignancies which originate from the oral cavity, pharynx, larynx, paranasal sinuses, and nasal cavity with more than 90% diagnosed as head and neck squamous cell carcinomas (HNSCCs) [1, 2]. Among all forms of fatal HNC, oral squamous cell carcinoma (OSCC) is the most common type of malignancy with 354,864 new cases diagnosed annually in the world [3]. In the US, the overall 5-year survival rate of OSCC patients was as high as 65%, but that of patients with advanced and recurred oral cancer was as low as 27% [4]. Currently, surgery with postoperative radiation- and/or chemotherapy remain the mainstay of treatment for patients with oral cancer [5, 6], but often causes severe morbidities that significantly affects patient quality of life [7, 8]. To date, several targeted drugs, such as epidermal growth factor receptor (EGFR) antibody cetuximab [9], and immune checkpoint inhibitors, such as programmed death receptor-1 (PD-1) antibodies nivolumab [10] and pembrolizumab [11], have been approved by FDA for the treatment of HNC. However, the overall response rate, particularly in patients with recurrent and metastatic (R/M) HNC is as low as 20% [12].

Tumor microenvironment (TME) is composed of complex cellular and noncellular components, including noncancerous stromal and immune cells [13, 14], that constitute complex communication networks via a large panel of bioactive molecules e.g., cytokines, chemokines, and growth factors [14]. Tumor-associated macrophages (TAMs) are among the most abundant type of cells in TME and constitute a link between the innate and adaptive immune responses, leading to the escape of cancer cells from immune surveillance [15–17]. A high density of TAMs has been shown in OSCC, which is consistently associated with poor prognosis [15, 18, 19]. Therefore, blocking TAM infiltration and polarization hold great promise for immunotherapy of cancer, particularly OSCC.

Polyamines, including putrescine, spermidine, and spermine, are essential for normal cell growth while elevated levels of polyamines are commonly observed in TME and have long been proved to be necessary for the transformation and progression of various types of cancers [20–22], thus rationally serving as potential targets for both therapy and prevention of cancer [20, 21, 23, 24]. The eukaryotic translation initiation factor 5 A (eIF5A), including two major isoforms, eIF5A1 and eIF5A2, acts as an important translation factor that functions at both the initiation and elongation phases of protein synthesis [25–27]. The major form, eIF5A1, is abundantly expressed in most types of normal cells and tissues, while eIF5A2 is undetectable but usually overexpressed in many types of cancer and closely associated with tumor growth, epithelial–mesenchymal transition (EMT), metastasis, recurrence, and poor prognosis [27]. Remarkably, eIF5A contains a unique amino acid hypusine that is formed by post-translational modification through transferring an aminobutyl group from spermidine to a specific lysine residue catalyzed by deoxyhypusine synthase (DHPS), leading to formation of deoxyhypusine, which is further converted to mature hypusine by deoxyhyposine hydroxylase (DOHH) [27]. DHPS and DOHH are two highly specialized enzymes that are not involved in other biochemical reactions other than eIF5A hypusination (eIF5A^hpu^) [27]. Spermidine-mediated hypusination appears to be essential to most, if not all, of eIF5A’s biological functions, thus rationally emerging as a potential target for both therapy and prevention of cancer [27, 28]. In addition to its important role in regulating several hallmarks of cancer cells, accumulating evidence has shown that polyamines also contribute to the establishment of an immunosuppressive TME through facilitating tumor infiltration and activation of immunosuppressive immune cells such as myeloid-derived suppressor cells (MDSCs), regulatory T cells (Tregs), and M2-like TAMs [29–34]. A recent study showed that eIF5A^hpu^ is essential to polyamine-induced M2 macrophage polarization [35]. However, to the best of our knowledge, no studies have ever explored the dual functions of eIF5A^hpu^ signaling axis in regulating tumor cell growth and TAM polarization in any type of cancer, particularly in OSCC.

In this study, we found an overall upregulation of *EIF5A2* mRNA and the transcripts of several key enzyme genes involved in polyamine metabolism, e.g., *ODC1*, *SRM*, and *SMOX*, in HPV^−^ HNSCC, and blocking DHPS/eIF5A^hpu^ consistently inhibited OSCC cell growth and the expression of genes involved in regulation of epithelial–mesenchymal transition (EMT) and cancer stem cell (CSC) properties both in vitro and in vivo. Meanwhile, we showed that blocking DHPS/eIF5A^hpu^ inhibited OSCC-induced M2-like polarization in vitro and tumor infiltration of M2-like TAMs in a syngeneic orthotopic murine OSCC model. These findings suggest elevated DHPS/eIF5A^hpu^ may play important dual functions in regulating cell proliferation/CSC properties of OSCC cells and M2-like TAM polarization, while eIF5A^hpu^ blocking therapy might represent a novel neoadjuvant treatment for patients with surgically resectable OSCC or synergize with immunotherapy for patients with recurrent and metastatic (R/M) OSCC.

## Results

### Polyamine-eIF5A^hpu^ axis-related genes were upregulated in human OSCC tissues

**Fig. 1:**
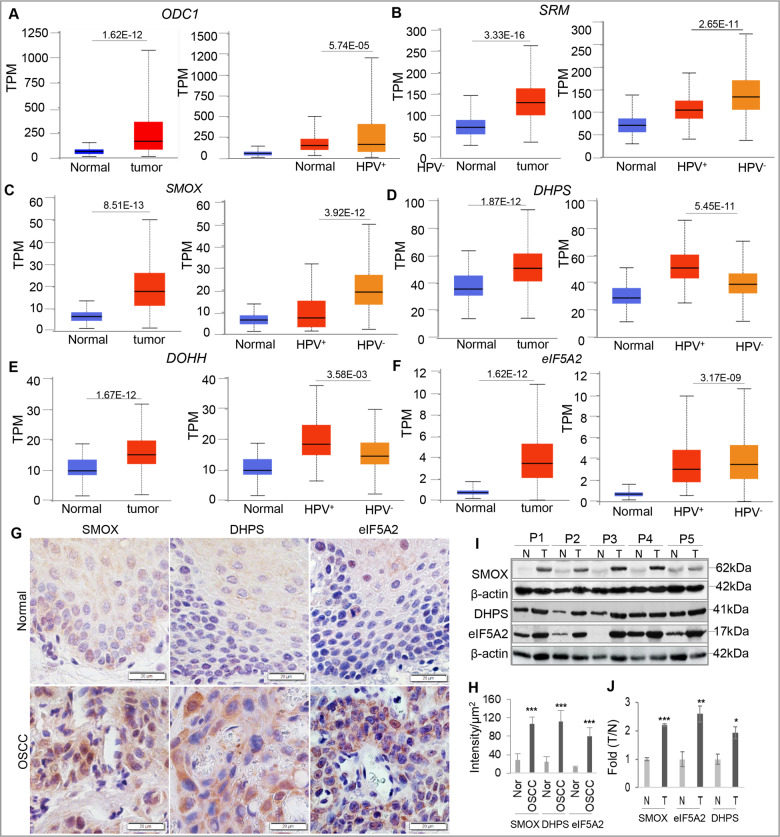
Upregulation of polyamine-eIF5A^hpu^ related gene expressions in OSCC tissues. **A**–**F** The expression of *eIF5A2* and several key enzyme genes involved polyamine metabolism and eIF5A hypusination, including *ODC1*, *SRM*, *SMOX*, and *DHPS*, in human HNSCC tissues (*n* = 520) and adjacent normal tissues (*n* = 44), was evaluated through TCGA dataset analysis using the UALCAN online tool. TPM transcript per kilobase million. **G** representative IHC staining for SMOX, DHPS, and eIF5A2 in OSCC tissues and adjacent normal tissues (scale bar = 20 μm). **H** Semiquantitative analysis of the IHC staining intensity using ImageJ software. ***P < 0.001, OSCC tissues versus normal tissues (mean ± SD). **I** The expression levels of SMOX, DHPS, and eIF5A2 proteins in five paired normal adjacent tissues (N) and OSCC tumor tissues (T) from the same patient (P1, P2, P3, P4, P5) were determined by western blot analysis, wherein β-actin was used as the loading control. **J** Semiquantification of protein band densities normalized to β-actin from the western blot analysis (**I**). The graph represents the fold change in the protein expression of individual genes in tumor tissues (T) as compared to that in normal tissues (N). *P < 0.05, **P < 0.01, ***P < 0.001 (mean ± SD; *n* = 5).

Due to the important role of polyamine-eIF5A^hpu^ axis in tumorigenesis and progression of a variety of cancers [27, 36], we initially performed TCGA data analysis using the UALCAN online tool to evaluate the expression of *EIF5A2* and several key enzyme genes involved polyamine metabolism, including *ODC1*, *SRM*, *SMOX*, *DHPS*, and *DOHH*, in human HNSCC tissues (*n* = 520) and adjacent normal tissues (*n* = 44). The results showed that the mRNA expression of *ODC1*, *SRM*, *SMOX*, *DHPS, DOHH*, and *EIF5A2* genes was significantly upregulated in OSCC tissues compared with that in adjacent normal tissues (Fig. 1A–F). Of note, the expression level of *EIF5A2, ODC1*, *SRM*, and *SMOX* mRNA was significantly higher in HPV^-^ HNSCCs than that in HPV^+^ HNSCCs (Fig. 1A–F). Meanwhile, a higher expression level of these genes was observed in Stages 1–4 of HNSCCs than that in normal tissues, among which *SMOX* and *DHPS* mRNA expression was higher in advanced HNSCC (stage 3 and stage 4) than that in lower stages of HNSCC (stage 1 and stage 2) (Supplementary Fig. 1A–F). In addition, a higher expression level of *ODC1* and *DPHS* mRNA was observed in relatively high grades of HNSCC than that in low grades of HNSCC (Supplementary Fig. 1A–F). Consistent with data from TCGA analysis, an elevated expression of SMOX, DHPS, and eIF5A2 at the protein level in OSCC tissues versus adjacent normal tissues was confirmed by immunohistochemical (IHC) (Fig. 1G, H) and Western blot (Fig. 1I, J and Supplementary File 1), respectively. These results support the notion that the polyamine-eIF5A^hpu^ axis may also play a role in the development and progression of HNSCCs.

### Blocking eIF5A^hpu^ inhibits the proliferation of OSCC cells

**Fig. 2:**
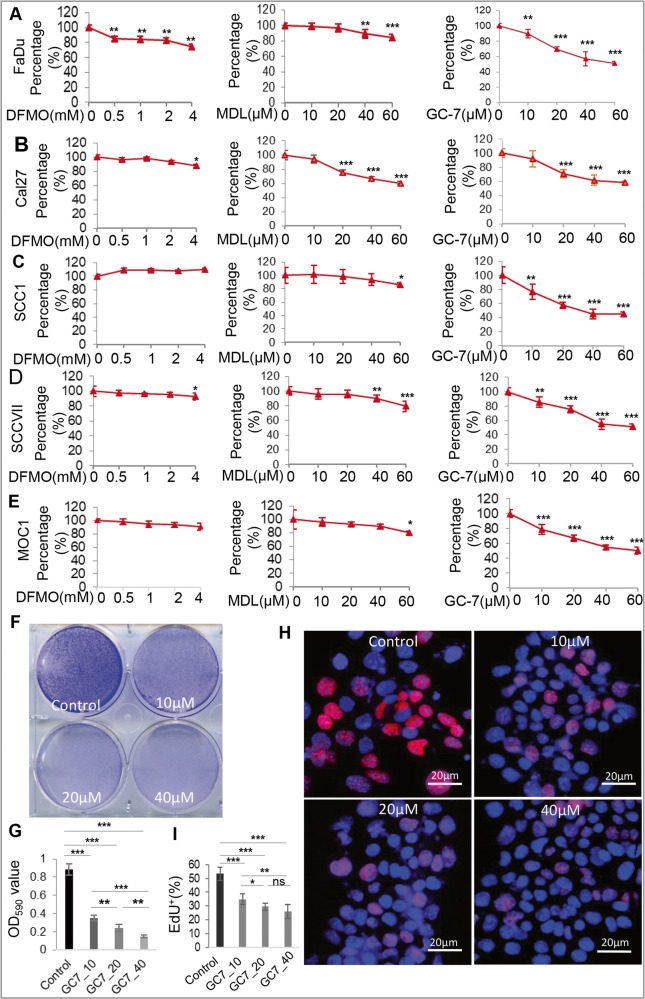
Blocking eIF5A^hpu^ inhibits proliferation of OSCC cells. **A**–**E** Human (FaDu, Cal27, and SCC1) and murine (SCC VII and MOC1) OSCC cells were treated with increasing concentrations of either DFMO (2-difluoromethylornithine), or MDL-72527 [N1, N4-bis (2,3-butadienyl)−1,4-butanediamine], or GC-7 for 48 h and the cell viability was determined by MTT assay, while cells without treatment were served as controls. *P < 0.05; **P < 0.01; ***P < 0.001 as compared with controls (designated as 100%). **F** FaDu cells were treated with different concentrations of GC-7 for 48 h and cell colonies were stained with 0.5% crystal violet staining solution. **G** Quantitative analysis of the crystal violet-stained cells, which were dissolved in 0.1 M sodium citrate solution (pH 4.0) and subjected to the measurement of absorbance at 590 nm. Each assay condition was done in triplicate. **H**, **I** Following treatment with different concentrations of GC-7 for 48 h, the incorporation of EdU (red) in FaDu cells was observed under a fluorescence microscope. The nuclei were counterstained with DAPI. For semiquantitative analysis, cells with positive EdU signals in at least six random fields were counted and expressed as the percentage of the total DAPI^+^ cell number. GC7_10, GC7_20, and GC7_40 represent 10, 20, and 40 µM of GC-7, respectively. Scale bars, 20 μm. *P < 0.05; **P < 0.01; ***P < 0.001; ns no significant difference.

Next, we determined the role of polyamine-eIF5A^hpu^ axis in the regulation of cell proliferation of OSCC cells. To this purpose, human Cal27, FaDu, and SCC1 cells and murine SCC-VII and MOC1 cells, were treated with increasing concentrations of either DFMO, MDL-72527, or GC-7 for 48 h, respectively. The results showed that blocking ODC1 activity by DFMO inhibited the proliferation of FaDu cells in a dose-dependent manner but had little effects on other OSCC cells (Fig. 2A–E, the left panel). Treatment with MDL-72527, a specific inhibitor of SMOX, dose-dependently inhibited the proliferation of Cal27 cells but significantly suppressed the proliferation of FaDu and SCC-VII cells at a relatively high concentration (≥40 µM) (Fig. 2A–E, the middle panel). Consistently, treatment of GC-7, a specific blocker of eIF5A^hpu^, robustly inhibited cell proliferation in a dose-dependent manner in all tested human and murine OSCC cell lines (Fig. 2A–E, the middle panel). Morphologically, treatment of OSCC cells with of GC-7 remarkably inhibited the colony formation even at a relatively low concentration (Fig. 2F, G and Supplementary Fig. 2). In addition, EdU incorporation assay indicated that treatment of FaDu cells with different concentrations of GC-7 for 48 h led to dose-dependent reduction in the proportion of EdU^+^ OSCC cells compared to the non-treatment control (*P* < 0.001) (Fig. 2H, I). These findings demonstrate that direct blockade of eIF5A^hpu^ resulted in a robust and reproducible inhibition of cell proliferation of OSCC cells, suggesting that eIF5A^hpu^ is a promising molecular target for the development of targeted therapy of OSCC.

### Potential molecular mechanisms underlying eIF5A^hpu^ blockade-mediated inhibition in the proliferation of OSCC cells

**Fig. 3:**
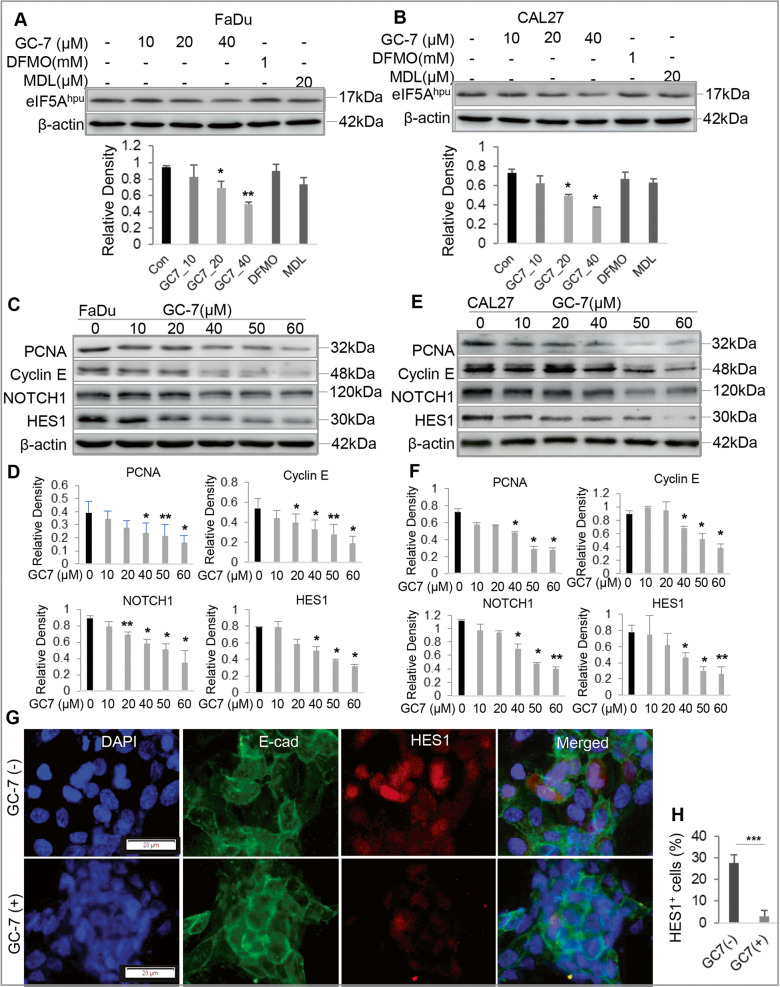
Blocking eIF5A^hpu^ downregulated the expression of proliferation-related genes in OSCC cells. **A**, **B** The downregulation of eIF5A^hpu^ in human OSCC cells (FaDu and Cal27) following treatment with different concentrations of GC-7 for 48 h was confirmed by western blot analysis, whereas blocking ODC1 activity by DFMO or blocking SMOX activity by MDL-72527 had minimal inhibitory effects on eIF5A^hpu^ in human OSCC cells. The lower panels of histograms represent the semiquantification of protein band densities normalized to β-actin from the western blot analysis. **C**, **E** Treatment with GC-7 for 48 h led to a dose-dependent downregulation in the protein expression of proliferating cell nuclear antigen (PCNA), Cyclin E, NOTCH1, and HES1 in human OSCC cells as determined by western blot. **D**, **F** Representative semiquantification of protein band densities normalized to β-actin from the western blot analysis (**C**, **E**). *P < 0.05, **P < 0.01 (mean ± SD). **G** The downregulation of HES1 protein expression in FaDu cells following treatment with 20 μM GC-7 for 48 h was determined by dual-color immunofluorescence staining (green, E-cadherin; red, HES1) and observed under a fluorescence microscope. The nuclei were counterstained with DAPI. Scale bars, 20 μm. **H** HES1^+^ cells in at least six random fields were counted and expressed as the percentage of the total DAPI^+^ cell number. ***P < 0.001 (mean ± SD).

Using a specific antibody for hypusinated eIF5A, we performed Western blot analysis and confirmed the specific inhibitory effect of GC-7 on eIF5A^hpu^ expression in human OSCC cells (Fig. 3A, B and Supplementary File 1). Of note, treatment with either DFMO or MDL-72527 had minimal inhibitory effects on eIF5A^hpu^ in OSCC cells (Fig. 3A, B). Correspondingly, GC-7 treatment led to a dose-dependent downregulation in the expression of proliferating cell nuclear antigen (PCNA) and cyclin E (Fig. 3C–F and Supplementary File 1), but not cyclins A, B, and D (data not shown), in OSCC cells. Since several lines of evidence have implied the important role of activation of NOTCH1 signaling pathway in tumorigenesis, cell proliferation, cancer stem cell formation and maintenance, and progression in OSCC [36–38], we then determined whether GC-7 treatment affects the expression of NOTCH1 and HES1, a downstream transcription factor of NOTCH signaling. The results showed that GC-7 treatment remarkably downregulated NOTCH1 and HES1 protein expression in OSCC cells as determined by western blot (Fig. 3C–F and Supplementary File 1) and IF staining (Fig. 3G, H). These findings suggest that blocking eIF5A^hpu^ by GC-7 inhibits cell proliferation of OSCC cells possibly by inactivation of the NOTCH1/HES1 signaling pathway.

### Blocking eIF5A^hpu^ suppressed the expression of genes involved in the regulation of EMT and CSCs

**Fig. 4:**
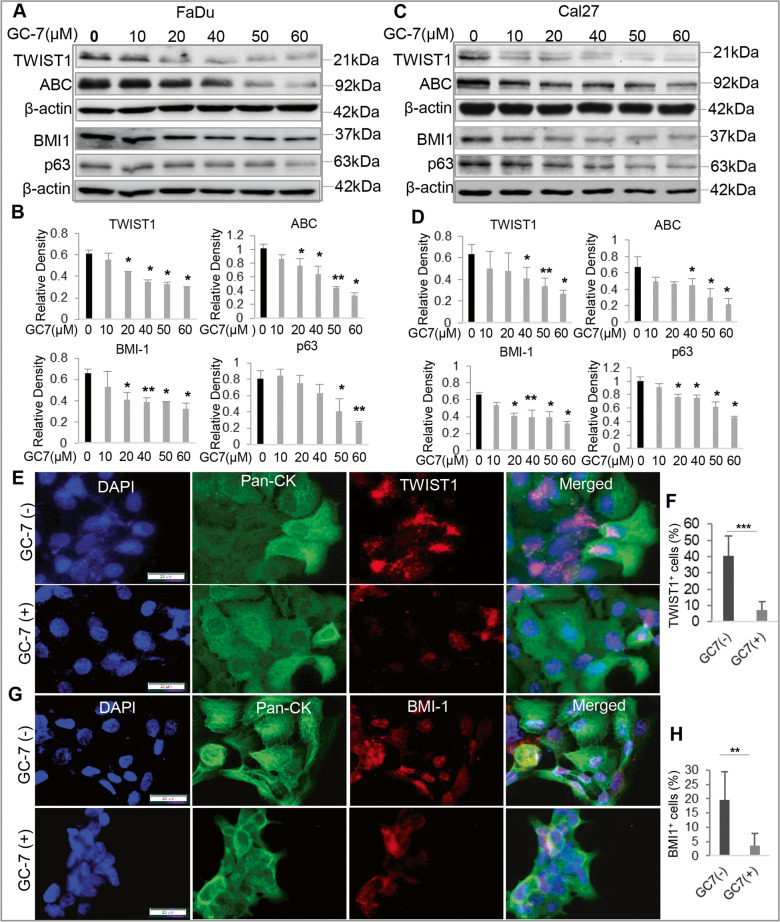
Blocking eIF5A^hpu^ inhibited the expression of genes involved in regulating EMT and CSC properties in OSCC cells. **A**, **C** Treatment with GC-7 for 48 h led to a dose-dependent downregulation in the protein expression of TWIST1, the active β-catenin (ABC), BMI-1, and p63 in human OSCC cells (FaDu and Cal27) as determined by western blot. **B**, **D** Representative semiquantification of protein band densities normalized to β-actin from the western blot analysis (**A**, **C**). *P < 0.05, **P < 0.01 (mean ± SD). **E**, **G** The downregulation of TWIST1 and BMI-1 protein expression in FaDu cells following treatment with 20 μM GC-7 for 48 h was determined by dual-color immunofluorescence staining (green, Pan-CK; red, TWIST1 or BMI-1) and observed under a fluorescence microscope. The nuclei were counterstained with DAPI. Scale bars, 20 μm. **F**, **H** TWIST1^+^ and BMI-1^+^ cells in at least six random fields were counted and expressed as the percentage of the total DAPI^+^ cell number. **P < 0.01^,^ ***P < 0.001 (mean ± SD).

Epithelial–mesenchymal transition (EMT) plays an important role in the formation and maintenance of cancer stem cells (CSC) in various types of malignancies [39], including OSCC [40, 41]. TWIST1, a master regulator of EMT program, has been shown to directly govern the expression of BMI-1, a key transcription factor involved in the regulation CSC properties in HNSCC [42], while recent studies have shown that eIF5A2 plays an important role in EMT in OSCC cells [43, 44]. We then asked whether blocking eIF5A^hpu^ suppressed the expression of genes involved in the regulation of EMT and CSCs. As expected, treatment of OSCC cells with GC-7 led to a dose-dependent downregulation in the protein expression of EMT-/CSC-regulatory genes, including TWIST1, active β-catenin (ABC), BMI-1, and p63, as determined by western blot (Fig. 4A–D and Supplementary File 1) and immunofluorescence (IF) staining (Fig. 4E–H), respectively. To substantiate our findings, murine OSCC cells (MOC2) were transduced with specific mouse Dhps-shRNA lentiviral particles, and the knockdown of DHPS protein expression was confirmed by western blot (Supplementary Fig. 3A, B and Supplementary File 1). As expected, knockdown of *DHPS* gene expression robustly reduced eIF5A^hpu^, and concomitantly, the expression of ABC, BMI-1, and NOTCH1/HES1 in OSCC cells (Supplementary Fig. 3A, B and Supplementary File 1). These findings suggest that blocking eIF5A^hpu^ has the potential to suppress EMT and CSC properties in OSCC.

### Blocking eIF5A^hpu^ inhibited OSCC-induced polarization of M2-like tumor-associated macrophages (TAMs)

Previous studies have shown an increased infiltration of M2-like TAMs in OSCC, which is consistently associated with a poor prognosis [15, 18]. We then performed TCGA data analysis using the UALCAN online tool to evaluate the expression of monocyte/macrophage marker genes, including *MRC1* (CD206), *SCARI1*(CD163), *SCARD1* (CD68), *ITGAM* (CD11b), in HNSCC (*n* = 520) and adjacent normal (*n* = 44) tissues. The results showed that the mRNA expression of these genes was significantly upregulated in HNSCC tissues compared with that in adjacent normal tissues (Supplementary Fig. S4A–D). Meanwhile, a higher expression level of *SCARI1*(CD163), *SCARD1* (CD68), and *ITGAM* (CD11b) mRNA was observed in relatively high grades of HNSCC than that in low grades of HNSCC (Supplementary Fig. S4A–D). In addition, the expression level of *MRC1* (CD206) mRNA, a marker of M2 macrophages, was significantly higher in HPV^-^ HNSCCs than that in HPV^+^ HNSCCs (Supplementary Fig. S4A). These results further support the notion that increased infiltration of M2-like TAMs might play an important role in the development and progression of HNSCC, including OSCCs.

**Fig. 5:**
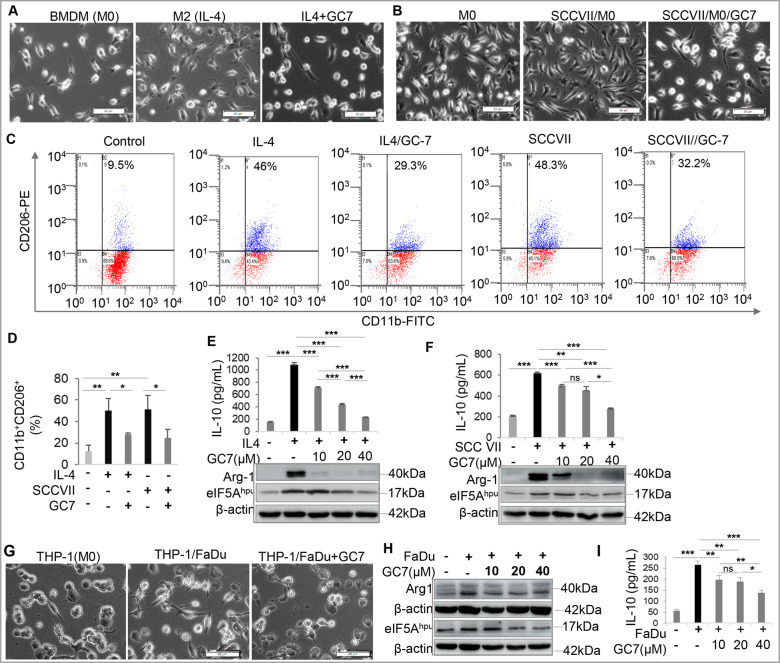
Blocking eIF5A^hpu^ inhibited OSCC-induced polarization of M2-like tumor-associated macrophages. **A**, **B** Murine bone marrow-derived macrophages (BMDMs) were stimulated with IL-4 (20 ng/mL) or co-cultured with murine SCC-VII cells (1:1) in the absence or presence of 20 μM GC-7 for 48 h and cell morphological changes were observed under a microscope. Scale bars, 50 μm. **C**, **D** The increased expression of CD206 in murine BMDMs induced by IL-4 stimulation or co-culture with SCC-VII cells was remarkably abrogated by GC-7 treatment as determined by flow cytometry (FCM), whereby the representative histograms of FCM (**C**) and the mean percentage (**D**) of CD11b^+^CD206^+^ cells (*n* = 3) were presented. **E** BMDMs were stimulated with IL-4 (20 ng/mL) in the absence or presence of different concentrations of GC-7 for 48 h. The secretion of IL-10 in the supernatant was determined by ELISA (the upper panel) and the expression level of eIF5A^hpu^ and arginase-1 (Arg1) was determined by western blot (the lower panels) while β-actin was used as the loading control. **F** BMDMs were co-cultured with murine SCC-VII cells (1:1) in the absence or presence of different concentrations of GC-7 for 48 h. The secretion of IL-10 in the supernatant was determined by ELISA (the upper panel) and the expression level of eIF5A^hpu^ and arginase-1 (Arg1) was determined by western blot (the lower panels) while β-actin was used as the loading control. **G** Human THP-1 macrophages co-cultured with FaDu cells (1:1) in the absence or presence of 20 μM GC-7 for 48 h and cell morphological changes were observed under a microscope. Scale bars, 50 μm. **H**, **I** THP-1 macrophages were co-cultured with FaDu cells (1:1) in the absence or presence of 20 μM GC-7 for 48 h. The secretion of IL-10 in the supernatant was determined by ELISA (**I**) and the expression level of eIF5A^hpu^ and arginase-1 (Arg1) was determined by western blot while β-actin was used as the loading control (**H**). *P < 0.05; **P < 0.01; ***P < 0.001; ns no significant difference.

We then explored whether blocking eIF5A^hpu^ by GC-7 has any effect on OSCC-induced polarization M2-like TAMs. Morphologically, we observed hallmark morphological changes of M2-like macrophages characterized by elongated projections [45] in murine bone marrow-derived macrophages (BMDMs) following stimulation with IL-4 or co-culture with murine SCC-VII cells for 48 h (Fig. 5A, B). However, such morphological changes induced by IL-4 or co-culture with SCC-VII cells were abrogated by the presence of GC-7 (Fig. 5A, B). Flow cytometric analysis showed that GC-7 treatment significantly reduced the increase in CD206 expression in murine BMDMs in response to IL-4 stimulation (from 50.13±11.19% to 28.07±1.5%) or co-culture with SCC-VII cells (from 51.33±13.02% to 24.7±7.76%), respectively (Fig. 5C, D). ELISA data showed that GC-7 treatment remarkably abrogated both IL-4 and SCC VII-induced increases in the secretion of IL-10 by BMDMs, one of the major anti-inflammatory cytokines secreted by M2 macrophages (Fig. 5E, F). Meanwhile, western blot analysis showed that GC-7 treatment almost completely abolished both IL-4 and SCC-VII-induced upregulation in the expression of arginase-1 (ARG1), a marker of M2 macrophages (Fig. 5E, F and Supplementary File 1). Of note, GC-7-mediated inhibitory effects on IL-4-induced M2 polarization and SCC-VII-induced polarization of TAMs correlates with its robust suppressive effect on IL-4- and SCC-VII-induced upregulation of eIF5A^hpu^ in murine BMDMs as determined by Western blot analysis (Fig. 5E, F and Supplementary File 1). The inhibitory effect of GC-7 on the mRNA expression of M2 macrophage-associated genes, e.g., *ARG1*, *CD206*, *IL-10*, and *TGF-β1*, in murine OSCC-induced TAMs was further confirmed by qRT-PCR (Supplementary Fig. 5).

To further confirm the inhibitory effect of GC-7 on OSCC-induced polarization of M2-like TAMs, human THP-1-derived M0 macrophages were co-cultured with FaDu cells in the presence or absence of different concentrations of GC-7 for 48 h. Similarly, blocking eIF5A^hpu^ by GC-7 abrogated OSCC-induced M2-like morphological changes (Fig. 5G). Correspondingly, human OSCC-induced upregulation in eIF5A^hpu^, ARG1 expression and IL-10 secretion in THP-1 macrophages was robustly attenuated by GC-7 treatment as determined by Western blot and ELISA, respectively (Fig. 5H, I and Supplementary File 1). The results from qRT-PCR showed that blocking eIF5A^hpu^ by GC-7 also robustly abrogated the upregulated mRNA expression of M2 macrophage-related genes, including *ARG1*, *CD163*, *CD206*, *IL-10*, and *TGF-β1*, in THP-1 macrophages induced by culture with human OSCC-derived conditioned medium (Supplementary Fig. 6). Taken together, these compelling results suggest that eIF5A^hpu^ plays a critical role in OSCC-induced M2-like polarization of TAMs.

### Blocking eIF5A^hpu^ suppressed OSCC tumor growth in vivo

**Fig. 6:**
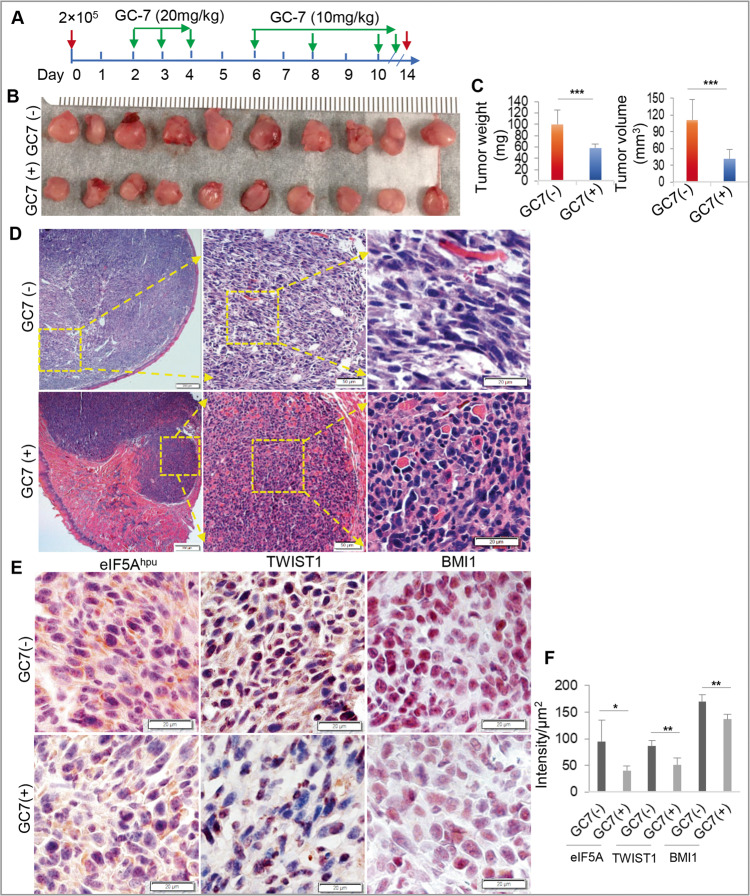
Blocking eIF5A^hpu^ retarded tumor growth in a syngeneic orthotopic tongue OSCC model in C3H mice. **A** murine SCC-VII cells (2 × 10^5^) in 20 µl of PBS were inoculated into the submucosa of the anterior tongue of C3H mice (*n* = 10). Two days later, GC-7 was administered according to the treatment regimen until day 14 post-transplantation. At the end of the experiments, tumors were dissected, weighed and tumor volumes were calculated accordingly. **B** Images of excised tumors from GC-7-treated and non-treated animals. **C** The mean value of tumor mass weights and volumes (mean ± SD; *n* = 10). **D** H&E staining of paraffin-embedded sections of murine OSCC tumor tissues. Scale bars: 20 μm (the right panel); 50 μm (the middle panel), and 200 μm (the left panel). The arrows indicate the partially enlarged views from the boxed areas. **E** representative images for IHC analysis on the expression eIF5A^hpu^, TWIST1, and BMI-1 in murine OSCC tumor tissues treated or non-treated with GC-7. Scale bar, 20 μm. **F** Semiquantitative analysis of the IHC staining intensity using ImageJ software. *P < 0.05; **P < 0.01, GC-7 treatment versus non-treatment (mean ± SD).

The compelling results from our in vitro studies encouraged us to further determine the potential role of eIF5A^hpu^ in OSCC growth in vivo. Using a syngeneic orthotopic tongue OSCC model in mice, we found that blocking eIF5A^hpu^ by GC-7 treatment significantly inhibited tumor growth, as evidenced by reduced tumor weight and volume as compared with the non-treatment control group (*P* < 0.001) (Fig. 6A–C). H & E staining showed that orthotopic OSCC tumor cells aggressively invaded the whole tongue tissue of the non-treated mice and had a histological appearance with large cell nuclei, irregular size and shape (Fig. 6D, the upper panel). On the contrary, in GC-7-treated mice, the tumor cells were less invasive into tongue muscles and loosely arranged with condensed nuclei, demonstrating that blocking eIF5A^hpu^ by GC-7 treatment significantly halted OSCC growth in vivo (Fig. 6D, the lower panel). In addition, immunohistochemical (IHC) studies showed that GC-7 treatment robustly inhibited the expression of TWIST1 and BMI-1 proteins, which complied with GC-7-mediated downregulation of eIF5A^hpu^ levels in xenografted tumor tissues (Fig. 6E, F). These findings suggest that eIF5A^hpu^ could be a novel therapeutic target for the treatment of OSCC.

### Blocking eIF5A^hpu^ reduced infiltration of M2-like TAMs in syngeneic orthotopic OSCC tumors

**Fig. 7:**
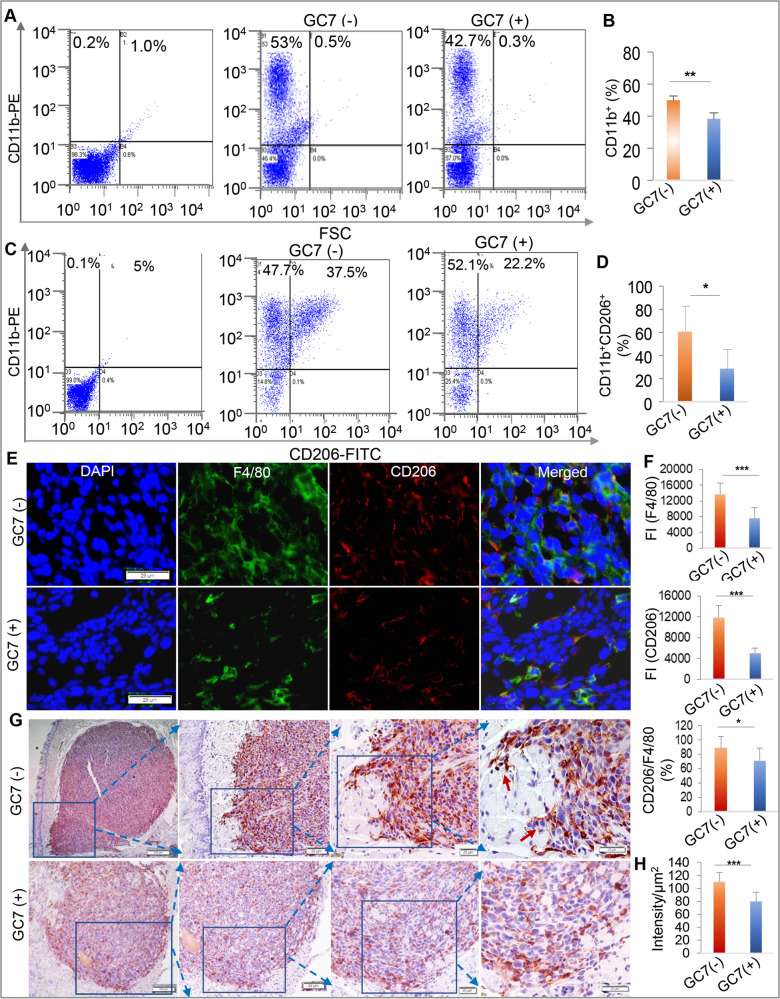
Blocking eIF5A^hpu^ reduced tumor infiltration of M2-like TAMs in syngeneic orthotopic murine tongue SCC tissues. **A**–**D** Blocking eIF5A^hpu^ by GC-7 treatment reduced the infiltration of total CD11b^+^ monocytes and CD11b^+^/CD206^+^ M2-like macrophages in murine OSCC tissues as determined by flow cytometric analysis, whereby the representative histograms of FCM (**A**, **C**) and the mean percentage of CD11b^+^ and CD11b^+^CD206^+^ cells (*n* = 3) were presented, respectively (**B**, **D**). **E** representative immunofluorescence staining for F4/80 (green) and CD206 (red) in murine OSCC tissues from GC-7-treated or non-treated mice. The nuclei were counterstained with DAPI. Scale bar = 20 μm. **F** Semiquantification of fluorescence intensity (FI) of F4/80 and CD206 as well as the IF ratio of CD206^+^ versus F4/80^+^ in murine OSCC tissues from GC-7-treated or non-treated mice. **G** Representative IHC staining for arginase-1 (Arg1) protein expression in murine OSCC tissues from GC-7 treated or non-treated mice. Scale bars, 200 μm (the first panel), 100 μm (the second panel), 50 μm (the third panel), 20 μm (the fourth panel). The blue arrows indicate the partially enlarged views of the boxed areas. The red arrows point to the intrusion/invasion of ARG1^+^ M2-like TAMs in mice tongue tissues. **H** Semiquantitative analysis of the IHC staining intensity using ImageJ software.*P < 0.05; **P < 0.01; ***P < 0.001, GC-7 treatment versus non-treatment (mean ± SD).

In addition to the potent inhibitory effect on OSCC cell growth both in vitro and in vivo, eIF5A^hpu^ blockade by GC-7 also robustly suppressed OSCC-induced polarization of M2-like TAMs in vitro. Meanwhile, we further showed that blocking eIF5A^hpu^ by GC-7 remarkably inhibited murine OSCC-induced increase in the migratory ability (Supplementary Fig. 7A) and CSF-1R mRNA and protein expressions (Supplementary Fig. 7B, C and Supplementary File 1) in murine Raw264.7. macrophages. We then evaluated the effect of blocking eIF5A^hpu^ by GC-7 on the infiltration of TAMs in the syngeneic orthotopic murine tongue SCC model. As a result, blocking eIF5A^hpu^ remarkably reduced tumor infiltration of total CD11b^+^ monocytes (Fig. 7A, B) and the proportion of CD206^+^ M2-like macrophages (Fig. 7C, D) in the orthotopic tongue tumor tissues compared to that in the non-treatment control group. Dual-color IF staining showed that blocking eIF5A^hpu^ by GC-7 treatment reduced tumor infiltration of total F4/80^+^ macrophages and the proportion of F4/80^+^/CD206^+^ macrophages (Fig. 7E, F) as well as CD206^+^/ARG1^+^ and CD206^+^/HO-1^+^ M2-like macrophages (Supplementary Fig. 8) in tumor microenvironment. Interestingly, we found that blocking eIF5A^hpu^ by GC-7 treatment obviously inhibited the intrusion/invasion (presented as “finger-like structures”) of ARG1^+^ M2-like TAMs in tongue tissues (Fig. 7G, H). These compelling results suggest that blocking eIF5A^hpu^ treatment could robustly attenuate infiltration of M2-like TAMs in syngeneic orthotopic OSCC tumors.

Since TAMs play an important role in the establishment of a “cold” immunosuppressive tumor microenvironment that contributes to immune escape of cancer cells, including HNSCC cells [46], while targeted inhibition of TAMs may lead to alterations in tumor immune profiles [47]. We them performed qRT-PCR to determine changes in the mRNA expression of a panel of immunosuppressive and inflammatory molecules in syngeneic orthotopic OSCC tumors following blocking eIF5A^hpu^ treatment with GC-7. Our results showed that blocking eIF5A^hpu^ downregulated the expression of several immunosuppressive molecules, including *Arg1*, *IL-10*, *RELMa*, *PD-1*, and *PD-L1*, and increased the expression of several pro-inflammatory molecules, including *CD86*, *TNF-α*, *CXCL9*, *CXCL10*, *IL-1b*, and *CCL5* (Supplementary Fig. 9). These results suggest that blocking eIF5A^hpu^ therapy may have the potential to improve the therapeutic efficacy of immunotherapies in OSCC patients.

## Discussion

eIF5A, particularly eIF5A2, is usually overexpressed in various types of cancer, while its activation due to spermidine-mediated hypusination through the action of two highly specialized enzymes, DHPS and DOHH, plays a critical role in regulating cell proliferation, apoptosis, EMT process, CSC properties, and metastasis of cancer cells [27, 48–51]. In the present study, we performed TCGA analysis of the HNSCC dataset and found that the mRNA expression of *EIF5A2* gene and several key enzyme genes involved in polyamine metabolism, including *ODC1*, *SRM*, *SMOX*, *DHPS*, and *DOHH*, was significantly upregulated in HNSCC tissues compared with those in adjacent normal tissues, which is positively correlated with disease stages and histological grades (Fig. 1 and Supplementary Fig. 1). HPV infection is emerging as an important risk factor for HNSCC, especially for oropharyngeal carcinoma, and HPV^−^ HNSCCs are usually more aggressive and have a worse prognosis than HPV^+^ HNSCCs [8, 52]. Herein, TCGA dataset analysis revealed that the mRNA expression levels of *ODC1*, *SRM*, *SMOX*, and *EIF5A2* genes are significantly higher in HPV^-^ HNSCC tissues than that in HPV^+^ HNSCC tissues (Fig. 1), suggesting that polyamine-eIF5A2 axis may play an important role in the aggressiveness and progression of HPV^-^ HNSCC. However, further studies are warranted to explore this scenario.

Polyamine blocking therapy (PBT), particularly, blocking ODC1 or SMOX activity, has shown promising therapeutic potentials in several types of cancer [20, 23, 24, 53]. Even though the expression of *ODC1 and SMOX* genes at both mRNA and protein levels is upregulated in HNSCC tissues (Fig. 1), blocking ODC1 activity by DFMO or SMOX activity by MDL-72527 does not consistently inhibit cell proliferation in tested human and murine OSCC cell lines (Fig. 2). On the contrary, pharmacologically blocking spermidine-dependent eIF5A^hpu^ through treatment with GC-7 results in consistent and robust inhibition in cell proliferation of all OSCC cell lines in vitro (Fig. 2). More convincingly, administration of GC-7 remarkably suppressed tumor growth in a syngeneic orthotopic murine OSCC model (Fig. 6). Most recently, a study has shown that high eIF5A2 expression is associated with an advanced N value and expression of EMT markers in human OSCC tissues and overexpression of eIF5A2 predicts an unfavorable prognosis in patients with OSCC [54]. Mechanistically, pharmacologically blocking eIF5A^hpu^ or specifically knocking down the expression of *Dhps* gene through transduction of Lenti-shRNA particles significantly downregulated the expression of cancer stem cell (CSC)-regulatory genes, e.g., active-β-catenin (ABC), BMI-1, and NOCTH1/HES1 signaling pathway in OSCC cells (Figs. 3 and 4 and Supplementary Fig. 3). Previous genomic analyses have identified *NOTCH1* gene is the second most frequently mutated gene after *TP53* in HNSCC, whilst most NOTCH1 mutations are reported to be inactive, suggestive of its tumor suppressor role [55]. However, numerous studies have also shown that NOTCH1 is upregulated in HNSCC tissues compared with adjacent normal tissues and plays positive roles in regulating tumor cell proliferation, EMT, invasion/metastasis, and CSC properties [36, 55–57]. These findings suggest that NOTCH1 plays a dual role, as a tumor suppressor and oncogene, depending on the context in HNSCC [55]. Herein, we showed that pharmacologically blocking eIF5A^hpu^ or genetic knockdown of *Dhps* gene expression remarkably inhibited NOTCH1/HES1 signaling in OSCC cells (Fig. 3 and Supplementary Fig. 3), suggesting that NOTCH1/HES1 signaling may function as the downstream targets of eIF5A^hpu^ involving in the regulation of cell proliferation and CSC properties of OSCC cells. However, further studies are warranted to explore the detailed mechanisms by which eIF5A^hpu^ regulates the activity of the NOTCH1/HES1 signaling pathway in OSCC cells.

In the complex tumor microenvironment (TME) [13, 14], M2-like tumor-associated macrophages (TAMs) possess anti-inflammatory and immunosuppressive activities, thus contributing to the establishment of an immunosuppressive TME that facilitates the escape of cancer cells from immune surveillance, and consequently, the aggressiveness and progression of malignancies [15–17]. Previous studies have shown that polyamines play an important role in regulating macrophage polarization and function [33], while polyamine blocking therapy (PBT) increased M1 macrophages in the TME of syngeneic murine ovarian cancer [32] but decreased immunosuppressive CD206^+^F4/80^+^ M2-like TAMs in syngeneic murine melanoma and colon cancers [31, 34]. Several lines of evidence have demonstrated that an increased density of TAMs, particularly those of a M2-like phenotype, correlate with advanced clinic stages, poor pathologic grades, and a poor overall survival and progression-free survival in HNSCC [15, 18, 19]. Through TCGA dataset analysis, we also found that the mRNA expression of several monocyte/macrophage marker genes, including *Mrc1* (CD206), *CD163*, *CD68*, and *ITGAM* (CD11b), was significantly upregulated in HNSCC tissues compared to that in adjacent normal tissues, which correlates with advanced clinical stages and poor pathological grades of HNSCC (Supplementary Fig. S3). Most recently, it has been shown that blocking eIF5A^hpu^ remarkably inhibits IL-4-induced classical M2 macrophage polarization [35], but little is known about the direct role of eIF5A^hpu^ in regulating the polarization of M2-like TAMs. In the present study, we showed that blocking eIF5A^hpu^ by GC-7 treatment dramatically inhibited the polarization and activation of M2-like TAMs induced by OSCC cells (Fig. 5 and Supplementary Figs. 5 and 6). In vivo, we showed that blocking eIF5A^hpu^ by GC-7 treatment significantly reduced the infiltration of total monocytes and M2-like TAMs in TME of a syngeneic orthotopic murine OSCC model (Fig. 7 and Supplementary Fig. 8). These findings suggest that targeting the immunosuppressive/pro-tumoral TAMs by blocking eIF5A^hpu^ might be a promising approach to improve the therapeutic outcome for HNSCC patients [58].

Polyamines contribute to the development of immunosuppressive TME not only by promoting M2-like TAM polarization but also by facilitating infiltration of immunosuppressive myeloid-derived suppressor cells (MDSCs), regulatory T cells (Tregs), and suppressing CD4^+^ and CD8^+^ T-cell functions [59]. Recent studies have shown that polyamine blocking therapy (PBT) reduced not only CD206^+^F4/80^+^ M2 macrophages but also tumor infiltrating cells including Gr-1^+^CD11b^+^ myeloid-derived suppressor cells (MDSCs) and CD4^+^CD25^+^ Tregs and concomitantly increased granzyme B^+^ and IFN-γ^+^ CD8^+^ T cells in TME [31, 34]. Meanwhile, it has been shown that PBT-mediated therapeutic effects on tumor growth were accompanied with reversing immunosuppression in TME and enhanced T-cell-dependent antitumor immunity [29, 30]. Most recently, a study has demonstrated that PBT significantly enhanced the antitumor efficacy of PD-1 blockade in both 4T1 and B16F10 tumors resistant to anti-PD-1 monotherapy, which was accompanied by reductions in MDSC and TAM subpopulations and an increase in tumor-specific cytotoxic T cells [34]. In the present study, we showed that blocking eIF5A^hpu^ by GC-7 treatment increased the expression of a panel of pro-inflammatory molecules and concomitantly reduced the expression of a panel of immunosuppressive molecules in syngeneic orthotopic murine OSCCs (Supplementary Fig. 9). These findings suggest that PBT and blocking eIF5A^hpu^ may be a common approach to boost antitumor immune responses. However, further studies are warranted to determine alterations in immune cell profiles and whether blockade of eIF5A^hpu^ can enhance immunotherapeutic efficacy in OSCC tumors.

In summary, to the best of our knowledge, our present study has shown for the first time that blocking eIF5A^hpu^ remarkably inhibits OSCC cell proliferation in vitro and syngeneic murine OSCC tumor growth in vivo. Meanwhile, blocking eIF5A^hpu^ also significantly inhibits the polarization and infiltration of OSCC-induced M2-like TAMs in TME of syngeneic orthotopic OSCC tumors. Our findings suggest that eIF5A^hpu^ may play a critical role in OSCC growth and progression due to its dual functions in regulating proliferation/CSC properties of OSCC cells and OSCC-induced M2-like TAM polarization, thus serving as a potential novel molecular target for prevention and treatment of OSCC.

## Materials and methods

### Human OSCC tissue collection

The study was conducted in accordance with human subject research guidelines and a protocol approved by the institutional review board (IRB) at the University of Pennsylvania (UPenn) (IRB#817407). Fresh tumor specimens diagnosed as oral squamous cell carcinomas and the corresponding adjacent normal were obtained immediately post-surgical procedures from the Department of Oral and Maxillofacial Surgery of Penn Medicine Hospital of UPenn. Informed consent was obtained from all subjects.

### Animals

Male C3H mice of 8-week-old were obtained from Charles River Laboratories. All animal studies were carried out in compliance with the guidelines of Institutional Animal Care and Use Committee (IACUC) of University of Pennsylvania. We followed a randomized, prospective, and controlled animal model design according to all the recommendations of the ARRIVE (Animal Research: Reporting In Vivo Experiments) guidelines. Mice were group-housed in polycarbonate cages (five animals per cage) in the animal facilities with controlled temperature (23 °C ± 2 °C), 40–65% humidity, and a 12-h light/dark cycle. Mice were acclimatized for at least 1 week before the study, fed with a standard laboratory diet, and allowed ad libitum access to drinking water.

### TCGA data analysis

The Cancer Genome Atlas (TCGA) expression data of polyamine metabolism-related enzymes, including ornithine decarboxylase 1(*ODC1*), spermidine synthase (*SRM*), spermine oxidase (*SMOX*), deoxyhypusine synthase (*DHPS*), eukaryotic translation initiation factor 5A2 (*EIF5A2*) as well as the expression of monocyte/macrophage marker genes, including *MRC1* (CD206), *SCARI1* (CD163), *SCARD1* (CD68), *ITGAM* (CD11b), and clinical data in HNSCC were downloaded from UALCAN databases (http://ualcan.path.uab.edu) [60]. All data are available online without any ethical and copyright conflicts. The expression levels of these genes in HNSCC (*N* = 520) and adjacent normal (*N* = 44) tissues were analyzed and correlated with clinicopathological parameters such as individual cancer stages, tumor grades, HPV status, and nodal metastasis.

### Cell culture

Cal27, a human tongue squamous cell carcinoma cell line, FaDu, a human squamous cell carcinoma cell line derived from the pharynx, were obtained from ATCC. Murine SCC-VII cells were kindly provided by Dr. Richard J Wong at Memorial Sloan Kettering Cancer Center (New York). Murine OSCC cell lines, MOC1 and MOC2, were obtained from Kerafast Inc. (Boston, MA). SCC1, an OSCC cell line derived from a recurrent squamous cell carcinoma of the floor-of-the mouth, was kindly gifted by Dr. Cun-yu Wang (University of California, Los Angeles). Cal27 and SCC-VII cells were cultured in DMEM supplemented with 10% fetal bovine serum (FBS, Zenbio), 100 U/mL penicillin, and 100 μg/mL streptomycin (Life Technologies). FaDu and SCC1 cells were cultured in RPMI-1640 medium containing with 10% FBS, 100 U/mL penicillin, and 100 μg/ml streptomycin. MOC1 and MOC2 cells were cultured in Iscove’s Modified Dulbecco’s Medium (IMDM; Corning)/Ham’s F12 (Corning) at a 2:1 mixture with 5% fetal bovine serum (Life Technologies), 1% penicillin/streptomycin (Life Technologies), 5 ng/ml epidermal growth factor (EGF; Peprotech), 400 ng/ml hydrocortisone (Sigma-Aldrich), and 5 μg/ml insulin (Sigma-Aldrich) according to the Kerafast’s protocol [61].

Human THP-1 monocyte and murine RAW264.7 cell lines were obtained from ATCC. THP-1 cells were cultured in RPMI-1640 medium containing with 10% FBS, 2-mercaptoethanol (0.05 mM), 100 U/mL penicillin, 100 μg/ml streptomycin. RAW264.7 cells were cultured in DMEM supplemented with 10% FBS, 100 U/mL penicillin, and 100 μg/ml streptomycin. Murine bone marrow-derived macrophages (BMDMs) were isolated and cultured as described previously [62]. Briefly, following euthanasia with CO_2_ inhalation, the femurs and tibias were separated from mice and bone marrow was flushed out and filtrated through 70-µm cell strainers. After centrifuging for 5 min at 1200 rpm, the pellet was resuspended in 2 mL red cell lysis buffer (BioLegend) for 3 min followed by the addition of PBS and centrifugation (1200 rpm, 5 min). Then, the pellet was resuspended in complete RPMI-1640 medium, nucleated bone marrow cells were seeded at 2 × 10^6^/well onto six-well tissue culture plates and incubated overnight, and non-attached cells were removed by washing with PBS. The attached cells were continuously cultured in complete RPMI-1640 medium containing 10 ng/mL murine recombinant M-CSF (BioLegend) for 6 days (M0 macrophage). Cell cultures were maintained in a humidified incubator with 5% CO_2_ at 37 °C.

### Cell viability and EdU incorporation assay

For the cell viability assay, human and murine OSCC cells were seeded into 96-well plates at 1 × 10^4^ cells per well in complete containing 5% FBS and cultured overnight. Subsequently, the culture medium was replaced with serum-free medium containing different concentrations of 2-difluoromethylornithine (DFMO, a specific ODC inhibitor), N1, N4-bis (2,3-butadienyl)−1,4-butanediamine (MDL-72527, a specific SMOX inhibitor), or GC-7 (CAYMAN Chemical Corp) for 48 h. Subsequently, 10 μL per well of MTT [3-(4,5-dimethylthiazol-2-yl)−2,5-diphenyltetrazolium bromide] solution (R&D systems) was added and incubated for 4 h. The medium was aspirated and replaced with 100 μL DMSO per well. The absorbance was measured at 590 nm using an OPSYS Mr microplate reader (Thermo Fisher Scientific). For the colony-forming assay, FaDu cells seeded in the six-well plate (1 × 10^5^/well) were treated with different concentrations of GC-7 for 48 h. Cell colonies were stained with 0.5% crystal violet staining solution, and the images were scanned by using Epson V700 Scanner. The crystal violet-stained cells were dissolved in 0.1 M sodium citrate solution (pH 4.0), and the absorbance at 590 nm was measured [63].

For the EdU incorporation assay, OSCC cells (2 × 10^4^ per well) plated in an eight-well chamber slide were treated with 20 µM of GC-7 for 48 h. Then, the Click-iT Plus EdU Cell Proliferation Kit with Alexa Fluor™ 594 dye (Cat. # C10639; Thermo Fisher Scientific) was utilized to image the incorporation of the modified thymidine analog EdU (5-ethynyl-2’-deoxyuridine, a nucleoside analog of thymidine) in proliferating cells according to the manufacturer’s protocol. Nuclei were counterstained with Hoechst® 33342 solution. Images were captured under a fluorescence microscope (Olympus IX73).

### Transduction with lentiviral vectors

MOC2 cells were plated at a density of 5 × 10^4^ cells per well in 12-well plates in the complete IMDM/F12 culture medium. After 24 h, cells were transduced with mouse Dhps-shRNA lentiviral particles with four unique 29mer target-specific shRNA (TL512451V) or a non-specific scrambled control Lentiviral particles (TR30021V) (OriGene Technologies, Inc., Rockville, MD) in the presence of 8 μg/mL polybrene. 24 h following transduction, the medium was replaced with 2 mL of fresh culture medium and cells were cultured for another 48 h. The expression of tagged green fluorescent protein (GFP) was observed under fluorescent microscopy, and transduced cells were passaged and selected by treatment with 4 µg/mL of puromycin.

### Co-culture of OSCC cells with macrophages

THP-1 monocytes were seeded into a six-well plate (1 × 10^6^ cells/well) and treated with 100 nM phorbol 12-myristate 13-acetate (PMA) for 6 h to allow them to attach and differentiate into M0 macrophages. The co-culture of OSCC cells and macrophages was performed in a trans-well system with culture inserts (membrane pore size of 0.4 μm) in a six-well plate, wherein PMA-differentiated THP-1 macrophages or murine BMDMs were seeded at the bottom and OSCC cells were seeded into the upper chamber of the trans-well, followed by co-culturing for 48 h in the presence or absence of different concentrations of N1-guanyl-1,7-diaminoheptane (GC-7, a specific DHPS inhibitor) (Cayman Chemicals).

### Western blot analysis

The cells were lysed in RIPA lysis buffer (Santa Cruz) supplemented with protease inhibitor cocktail (Santa Cruz), then ultrasonicated and incubated at 4 °C for 1 h. The lysates were centrifuged at 12,000 × *g* for 15 min. Protein concentration was measured using BCA protein assay kit (BioVision). An equal amount of proteins from each sample (50 μg) was separated on 8–12% polyacrylamide-SDS gels and electroblotted onto 0.2-μm nitrocellulose membranes (GE Healthcare). Membranes were blocked using 5% nonfat milk in TBST buffer for 1 h at room temperature and incubated with primary antibodies at 4 °C overnight. Thereafter, the membranes were washed three times with TBST buffer and incubated with horseradish peroxidase-conjugated secondary antibody at room temperature for 1 h. The primary and secondary antibodies are listed in Supplementary Table 1. Finally, the protein bands on the membranes were visualized by enhanced chemiluminescence (ECL) detection and scanned using an Amersham Imager 680 (GE Healthcare). The integrated density of the bands was quantitatively analyzed using ImageJ software, wherein the band of β-actin was used as an internal loading control.

### Migration assay

The migration assay was conducted using a trans-well chamber (8 μm pore size; CELLTREAT) in a 24-well plate. RAW264.7 cells were seeded into the upper chambers of trans-wells (5 × 10^4^ cells/ well) with 100 μL complete DMEM medium. SCC-VII cells were seeded into the lower chambers (1.6 × 10^5^ cells /well) with 600 μL serum-free DMEM medium. GC-7 (0, 10, 20, 40 μM) was added to the upper and lower compartments at the same concentration. Following culture for 24 h, the trans-well chambers were gently washed with PBS twice and non-migrated cells were removed with a cotton-tipped swab. The cells on trans-wells were fixed with methanol for 20 min. Then the cells were stained by 0.1% crystal violet in room temperature for 20 min, and then gently washed with PBS. After air dry overnight, the migrated cells were photographed using a light microscope.

### Immunofluorescence analysis

Mice tumor tissues were fixed in 4% PFA overnight at 4 °C followed by embedded in OCT compound for frozen sections (10 μm-thickness). Cultured cells fixed with 4% PFA or frozen sections were permeabilized in 0.5% Triton X-100 in PBS for 20 min, and then blocked in 2.5% goat serum in PBS at room temperature for 1 h, followed by incubation with the primary antibodies overnight at 4 °C. After washing with PBS, samples were incubated with appropriate secondary antibodies at room temperature for 1 h. Nuclei were counterstained with 4′, 6-diamidino-2-phenylindole (DAPI, Abcam). The primary and secondary antibodies are listed in Supplementary Table 1. Images were captured using Olympus inverted fluorescence microscope (IX73). Isotype-matched control antibodies were used as negative controls. For semiquantitative analysis, cells with positive signals in at least six random fields were selected and quantified by Olympus cellSens software.

### Histology and immunohistochemical analysis

Human OSCC or syngeneic mice tumor tissues were fixed with 4% PFA. For histological study, paraffin-embedded sections were stained with H&E. For immunohistochemical studies, the paraffin-embedded sections (5 μm) were deparaffinized with xylene, rehydrated with graded ethanol, and heated in 10 mmol/L sodium citrate buffer (pH 6.0) for antigen retrieval. After blocking with 2.5% goat serum in PBS, the sections were incubated overnight at 4 °C with primary antibodies, then detected using the universal immunoperoxidase ABC kit. The primary and secondary antibodies are listed in Supplementary Table 1. All the sections were counterstained with hematoxylin. Images were captured using a light microscope (Olympus, IX73) and semiquantification was performed using ImageJ software.

### Real-time PCR

Total RNA was extracted from cells and tumor tissues using TRIzol reagent (Invitrogen). Nanodrop 2000 was used to determine the RNA concentration. Reverse transcription was carried out with 1 μg of RNA in a BIO-RAD thermal cycler, using a high-capacity cDNA reverse transcription kit (ABI). Quantitative polymerase chain reaction (qPCR) was performed in CFX96 Real-Time PCR System (Bio-Rad) using SYBR Green Master Mix (Qiagen). Reference gene β-actin was used as an internal control. The primer sequences are listed in Supplementary Table 2. Data analysis was performed using the 2^−^ΔΔ^CT^ method for relative quantification. All the reactions were performed in triplicate.

### ELISA

The concentration of IL-10 in supernatants of cultured cells was detected using ELISA kits (BioLegend) according to the manufacturer’s instructions.

### Orthotopic syngeneic mouse model of OSCC

In total, 2 × 10^5^ SCC-VII cells in 20 μL PBS were injected directly into the submucosa of anterior tongue of C3H mice. Twenty mice injected with SCCVII were randomly divided into two groups: GC-7 treatment group and control group (*n* = 10). Two days after tongue injection, mice were intraperitoneally administered with GC-7 at 20 mg/kg for 3 consecutive days. Subsequently, mice were treated with GC-7 (10 mg/kg) every 2 days until 12 days after injection. On the fourteenth day, mice were sacrificed and tumors were removed and weighed, and prepared for further analysis. The tumor volume was calculated by using the formula volume = 0.5 × length × width^2^. During the experiment, the investigators were blinded to the group allocation of the animals and tumor measurement. Based on previous experimental observations, no statistical method was used to predetermine the sample size for in vivo experiment. No data were excluded from the analysis.

### Isolation of single cells from mouse tumors and flow cytometric analysis

Tumors were dissected, minced, and then enzymatically digested in PBS containing collagenase type II at a final concentration of 1 mg/ml (Sigma) at 37 °C for 40 min. Cell suspensions were filtered through a 70-µm cell strainer and then carefully layered on Percoll (70%/30%) (GE Healthcare, Piscataway, NJ, USA), and then centrifuged at 2000 rpm for 20 min at 4 °C [64]. The corresponding lymphomononuclear layer was harvested and washed once with PBS. Then, cells were resuspended in cell staining buffer (0.5% BSA in PBS with 2 mM EDTA) and incubated with PE-conjugated rat anti-mouse CD11b and FITC-conjugated rat anti-mouse CD206 antibodies or an isotype IgG control in the dark for 30 min at 4 °C. The cell samples were analyzed by BD FACSCalibur Flow Cytometer. Data were processed and analyzed by FlowJo software.

### Statistical analysis

All data are presented as mean ± standard deviation (SD) from at least three independent experiments. Differences between experimental and control groups were analyzed by two-tailed unpaired Student’s *t* tests using SPSS when the variance is similar between the groups. *P* value of less than 0.05 was considered statistically significant.

## Supplementary information

Supplementary materials

CDDIS-author contribution-CDDIS-23-1061

## Data Availability

The published article and its supplementary materials include all datasets generated and analyzed for the current study. Additional data are available from the corresponding author on reasonable request.
