# Supplementary material for: Targeted inhibition of eIF5Ahpu suppresses tumor growth and polarization of M2-like tumor-associated macrophages in oral cancer
Source: Cell Death Dis. 2023 Aug 31;14(8):579. doi: 10.1038/s41419-023-06109-z (PMC10471704; doi:10.1038/s41419-023-06109-z)

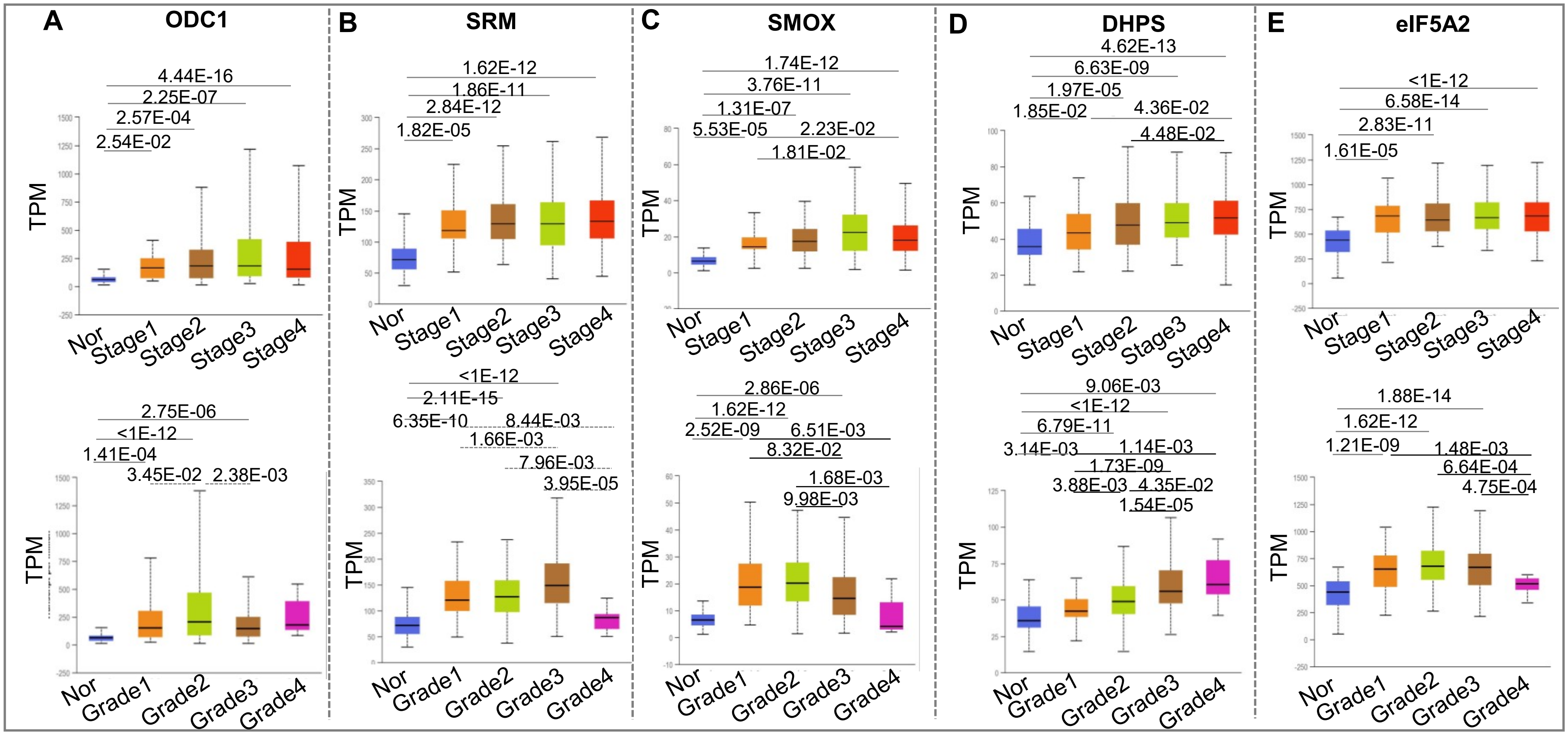

Supplementary Fig. 1. Upregulated expression of polyamine-eIF5A^{hpu} related genes correlates with the clinical stages and pathological grades of HNSCC. A-E (the upper panels), TCGA dataset analysis using the UALCAN online tool was performed to evaluate the correlation among clinical stages of HNSCCs (normal, n=44; stage 1, n=27; stage 2, n=71; stage 3, n=81; stage 4, n=264) and the expression levels of *eIF5A2* and several key enzyme genes involved polyamine metabolism and eIF5A^{hpu}, including *ODC1*, *SRM*, *SMOX*, and *DHPS* genes. TPM, transcript per kilobase million. **A-E** (the lower panels), TCGA dataset analysis using the UALCAN online tool was performed to evaluate the correlation among pathological grades of HNSCCs (normal, n=44; grade 1, n=62; grade 2, n=303; grade 3, n=125; grade 4, n=7) and the expression of *eIF5A2* and several key enzyme genes involved polyamine metabolism and eIF5A^{hpu}, including *ODC1*, *SRM*, *SMOX*, and *DHPS* genes. TPM, transcript per kilobase million.

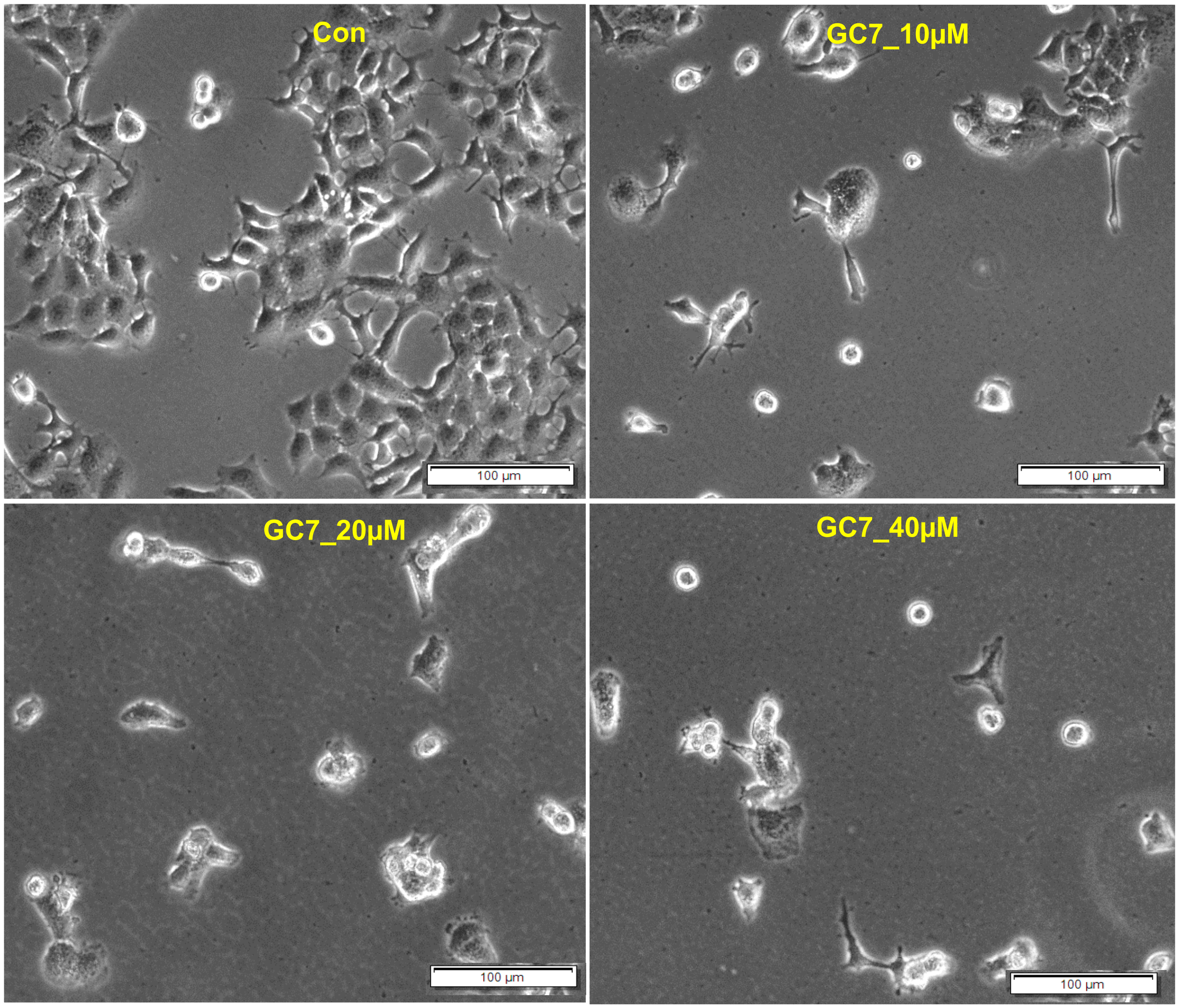

Supplementary Fig. 2. **Blocking eIF5A^{hpu} inhibits the proliferation of FaDu cells.** FaDu cells seeded in a 6-well plate (1×105/well) were treated with different concentrations of GC-7 (0, 10, 20, 40μM) for 48h and observed under a microscope. Scale bar, 100μm.

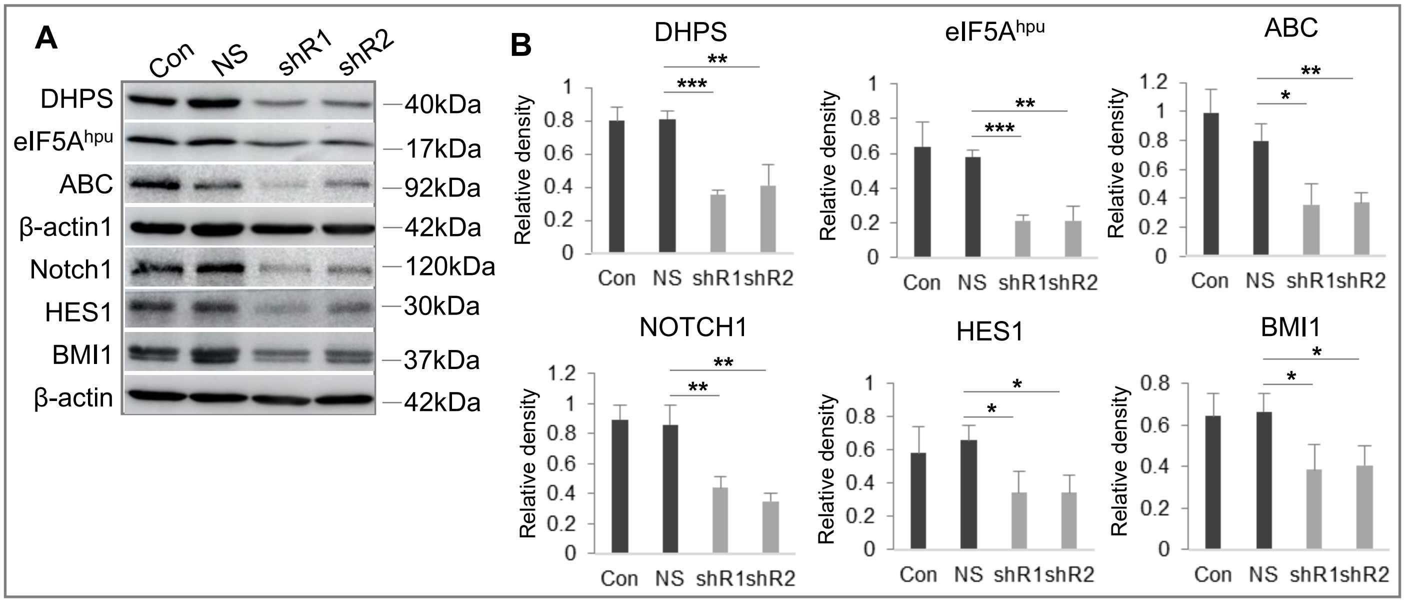

Supplementary Fig. 3. Knockdown of Dhps gene expression downregulated eIF5A^{hpu} and the expression of CSC-related genes in murine OSCCs. MOC2 cells were transduced with specific Dhps-shRNA lentiviral particles (shR1, shR2) or non-specific scrambled control lentiviral particles (NS) and selected with 4μg/mL of puromycin. **A**, the protein expression levels of DHPS, eIF5A^{hpu} active-β-catenin (ABC), NOTCH1, HES1, and BMI1 in MOC2 cells were determined by Western blot analysis. **B**, semi-quantification of protein band densities normalized to β-actin from the Western blot analysis. **P*<0.05; ***P*<0.01; ****P*<0.001 (mean ± SD).

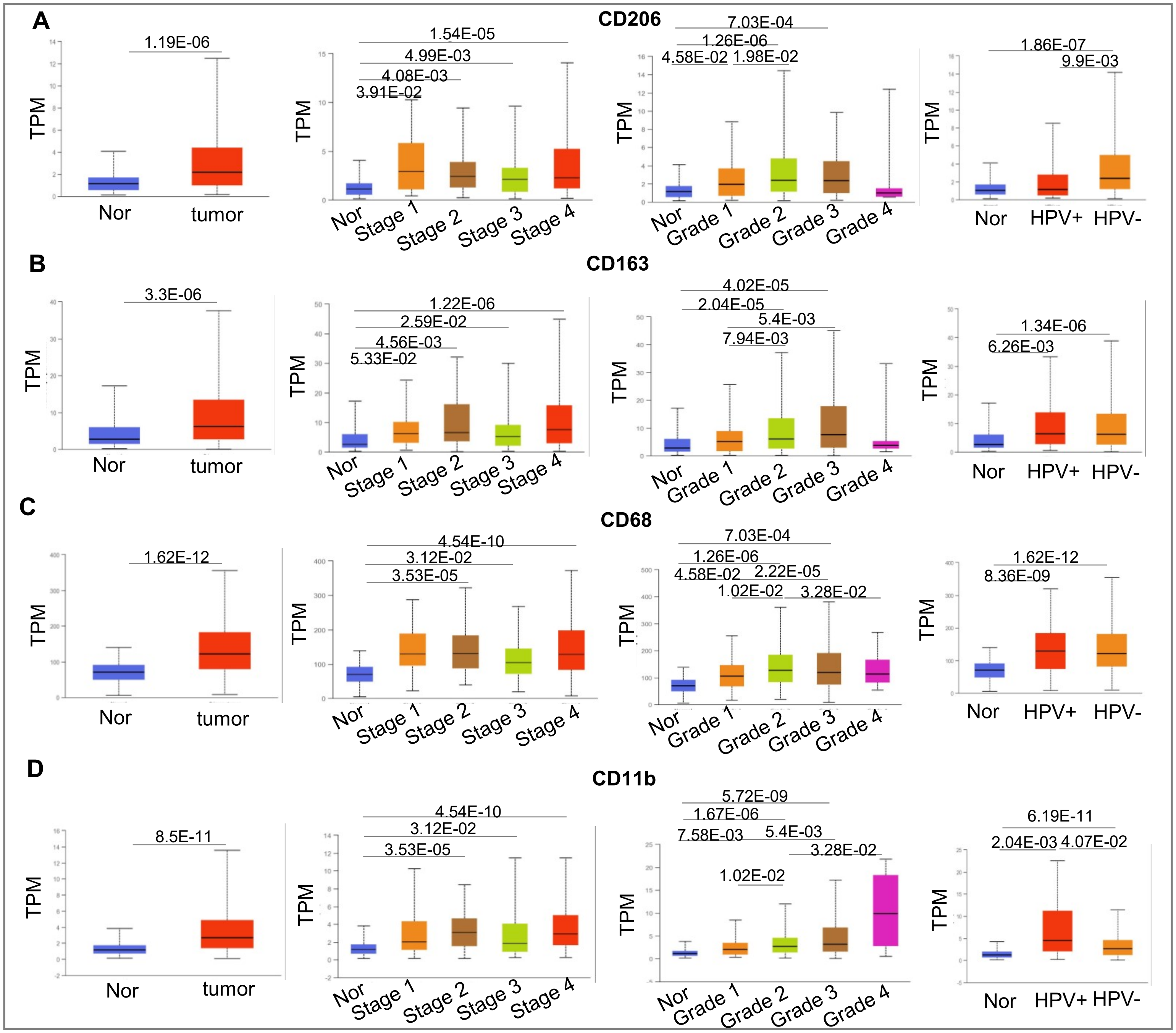

Supplementary Fig. 4. Upregulated expression of macrophage markers with the clinical stages and pathological grades of HNSCC. A-D, TCGA dataset analysis using the UALCAN online tool was performed to evaluate the correlation among clinical stages (normal, n=44; stage 1, n=27; stage 2, n=71; stage 3, n=81; stage 4, n=264), the pathological grades (normal, n=44; grade 1, n=62; grade 2, n=303; grade 3, n=125; grade 4, n=7), and HPV infection status (HPV^{+}, n=80; HPV^{-}, n=434) of HNSCCs with the expression levels of *CD206, CD163, CD68,* and *CD11b* genes, respectively. TPM, transcript per kilobase million.

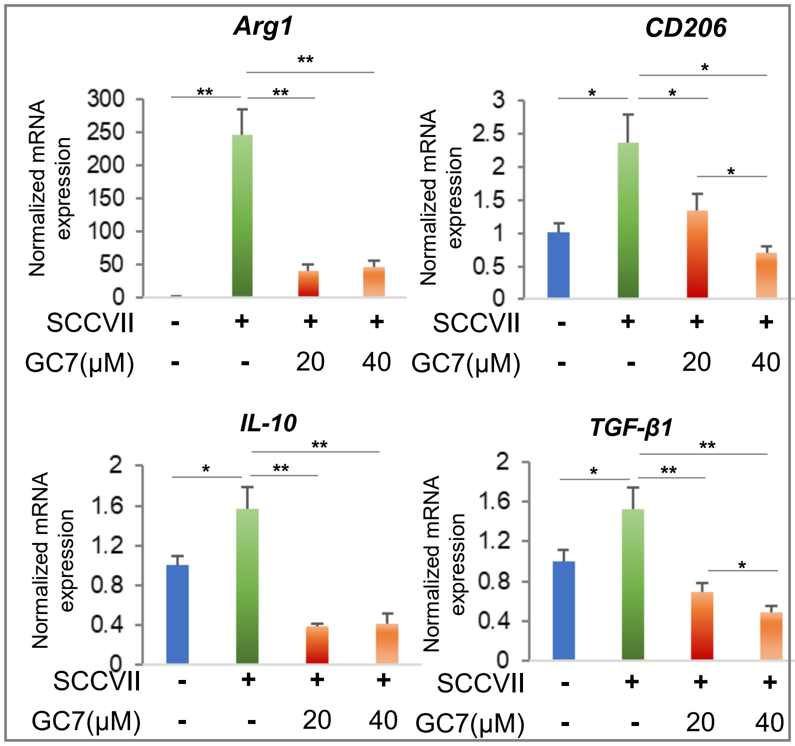

Supplementary Fig.5. Blocking eIF5A^{hpu} inhibits murine OSCC-induced M2-like TAM polarization. Mice bone marrow-derived macrophages were co-cultured with SCCVII cells in the presence or absence of GC-7 (20μM, 40μM) for 24h. The mRNA expression of M2 macrophage-associated genes was determined by qRT-PCR. *P<0.05; **P<0.01; ***P<0.001.

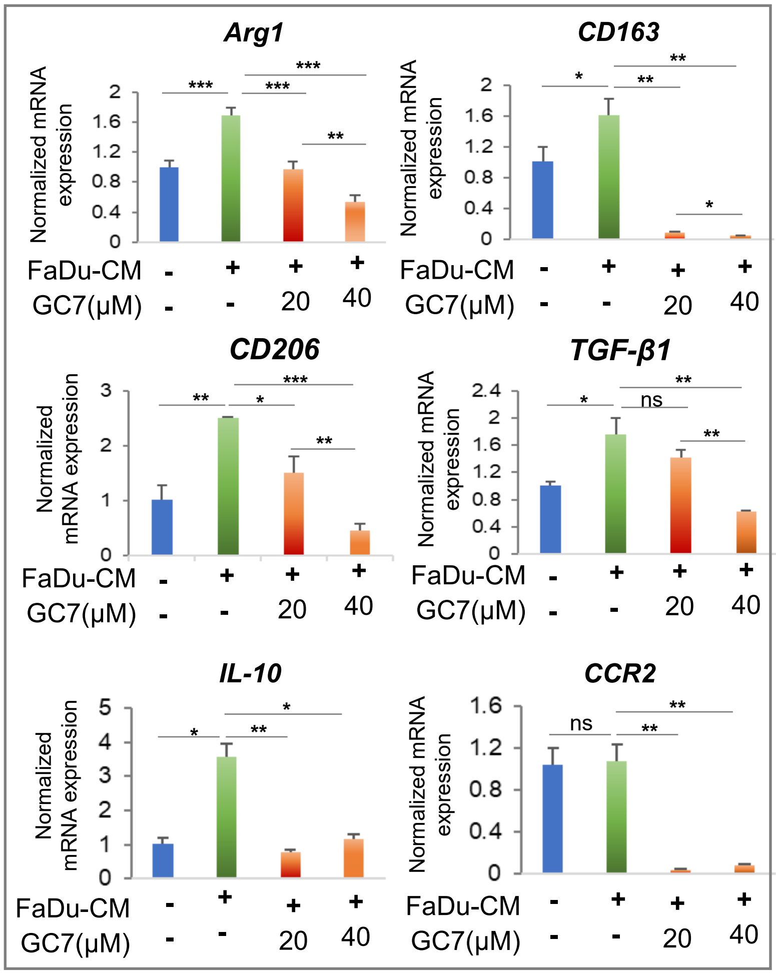

Supplementary Fig. 6. Blocking eIF5A^{hpu} inhibits M2 macrophage-related gene expressions in human OSCC-induced TAMs. THP-1 macrophages were cultured with FaDu-derived conditioned medium (1:1) in the absence or presence of 20μM and 40μM GC-7 for 24h. The mRNA expression level of *Arg1*, *CD163*, *CD206*, *IL-10*, *TGF-β1* genes was determined by qRT-PCR and the reference gene β-actin was used as an internal control. The experiments were performed in triplicates. **P*<0.05; ***P*<0.01; ****P*<0.001.

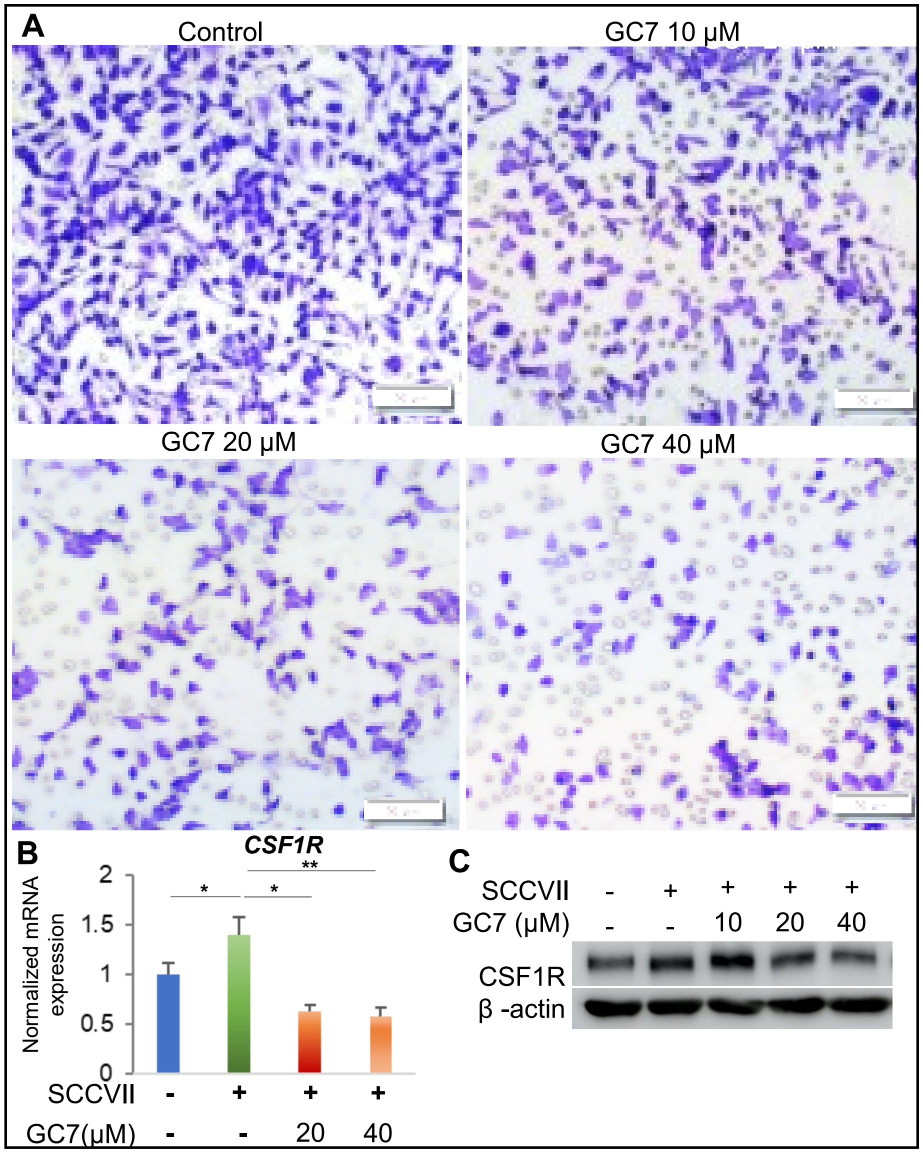

Supplementary Fig. 7. *Blocking eIF5A^{hpu} suppressed OSCC-stimulated migration of macrophages*. A, murine RAW264.7 cells and SCC-VII cells were co-cultured in the absence or presence of different concentrations of GC-7 (10, 20, 40μM) in a migratory assay system (with an insert with 8μm pores) for 24 h and the migrated cells were stained with Violet Crystal solution and photographed under a microscope. Scale bar, 50μm. **B** and **C**, murine BMDMs were cultured alone or co-cultured with SCC VII cells in the absence or presence of different concentrations of GC-7 (10, 20, 40μM) for 48 h and the expression of CSF1R mRNA and protein was assessed by qRT-PCR (B) and Western blot (C), respectively.

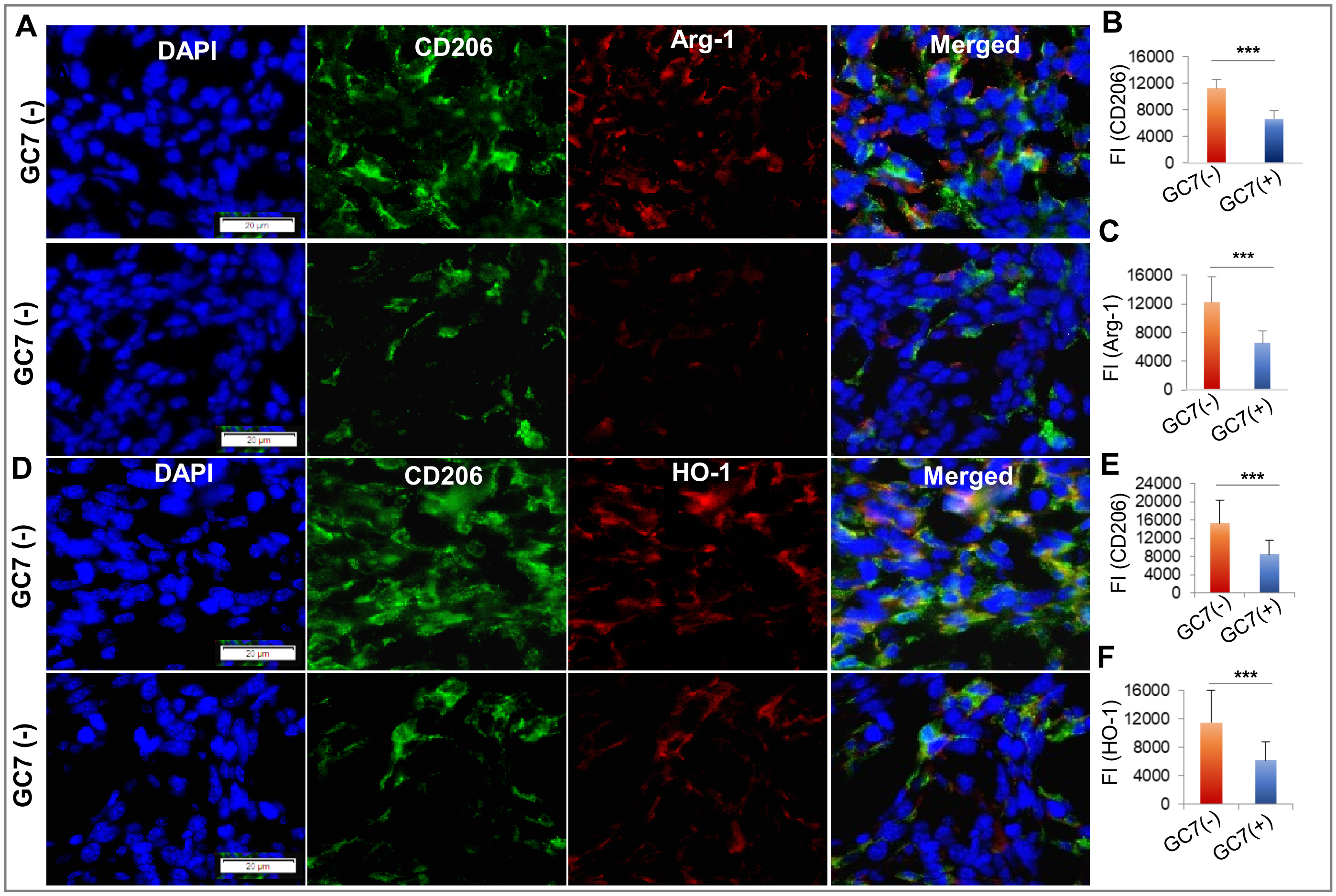

Supplementary Fig. 8. ***Blocking eIF5A^{hpu} reduced tumor infiltration of M2-like TAMs in orthotopic murine tongue OSCC tissues*.** **A**, treatment with GC-7 reduced the infiltration of Arg1^{+}/CD206^{+} M2-like macrophages in murine OSCC tissues as determined by dual-color immunofluorescence staining for CD206 (green) and arginase-1 (Arg-1, red) in murine OSCC tissues from GC-7 treated or non-treated mice. The nuclei were counter stained with DAPI. Scale bar = 20µm. **B** and **C**, semi-quantification of fluorescence intensity (FI) of CD206 and Arg-1. **D**, treatment with GC-7 reduced the infiltration of HO-1^{+}/CD206^{+} M2-like macrophages in murine OSCC tissues as determined by dual-color immunofluorescence staining for CD206 (green) and heme oxygenase-1 (HO-1, red) in murine OSCC tissues from GC-7 treated or non-treated mice. The nuclei were counter stained with DAPI. Scale bar = 20µm. **E** and **F**, semi-quantification of fluorescence intensity (FI) of CD206 and HO-1. ***P<0.001.

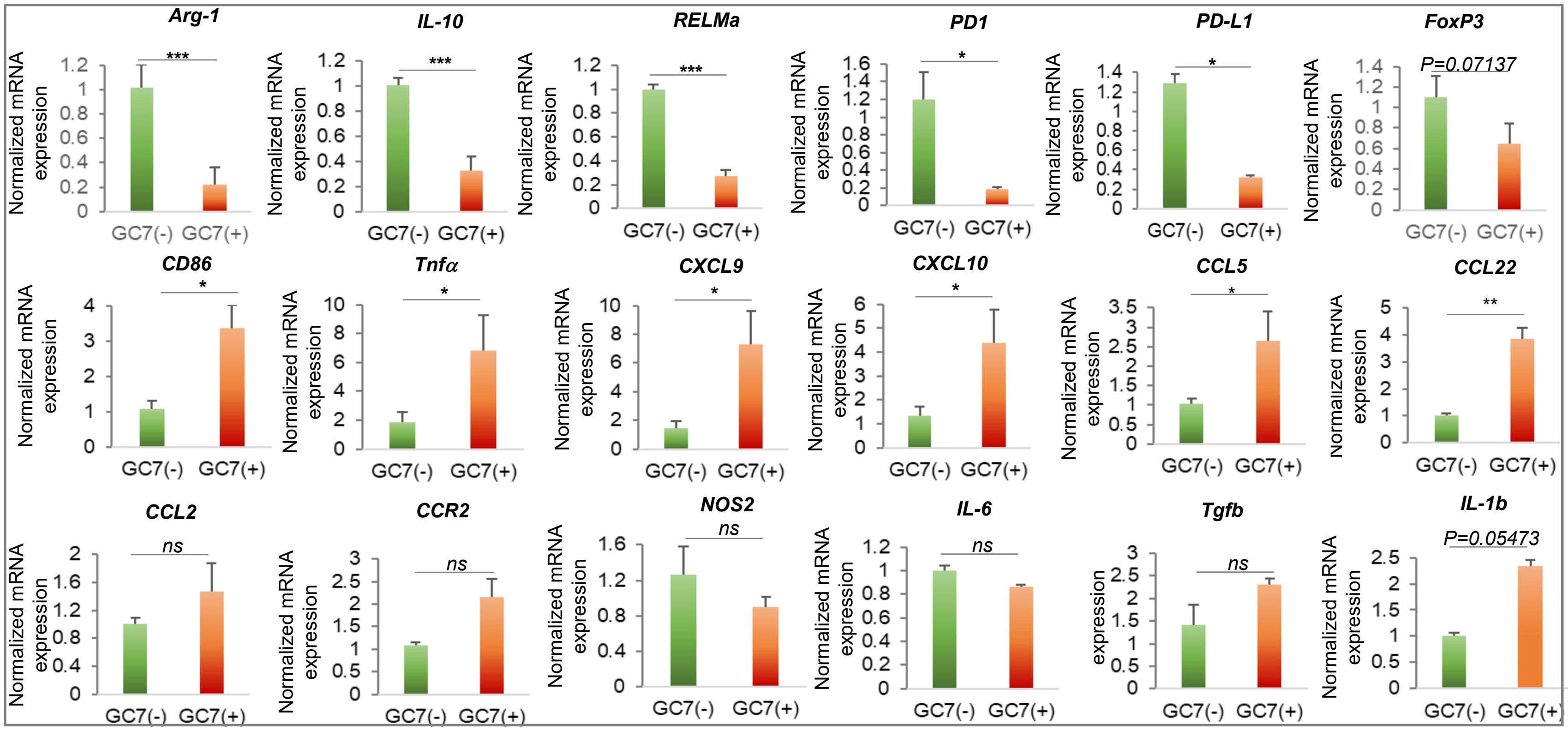

Supplementary Fig. 9. ***Blocking eIF5A^{hpu} altered the profiles of immune molecules in syngeneic orthotopic murine tongue OSCC.*** Total RNA was extracted from murine OSCC tumor tissues from GC-7 treated and non-treated mice. The mRNA expression of a panel of several immunosuppressive and inflammatory molecules was determined by qRT-PCR., including *Arg-1*, *IL-10*, *RELMa*, *PD1*, *PD-L1*, *FoxP3*, *CD86*, *TNF-α*, *CXCL9*, *CXCL10*, *NOS2*, *IL-6*, *CCL2*, *CCR2*, *CCL22*, *TGF-β*, *IL-1b,* and *CCL5* genes and the reference gene β-actin was used as an internal control. The experiments were performed in triplicates. * *P*<0.05; ***P* <0.01; ****P* <0.001.

Supplementary Table 1. Primary and secondary antibodies

Antibody name	Application	Company	Cat#
SMOX	WB (1:1000); IHC, IF (1:200)	ProteinTech	15052-1-AP
eIF5A2	WB (1:1000); IHC (1:200)	Lifespan Bioscicences	LS-c174144
Hypusinated eIF5A	WB (1:1000); IHC (1:200)	Creative Biolabs	PABL-202
DHPS	IHC (1:200)	ProteinTech	11184-1-AP
DHPS	WB (1:1000)	Abcam	ab202133
BMI-1	WB (1:1000); IHC, IF (1:100)	Santa Cruz	sc-10745
PCNA	WB (1:1000)	Santa Cruz	sc-7907
P63	WB (1:1000)	Abcam	Ab735
TWIST1	IHC, IF (1:200)	GeneTex	GTX127310
TWIST1	WB (1:1000)	Santa Cruz	sc-81417
Active β-catenin	WB (1:1000)	Cell Signaling Technology	8814S
HES1	WB (1:1000), IF (1:200)	Cell Signaling Technology	11988s
NOTCH1	WB (1:1000)	Cell Signaling Technology	3608s
Cyclin E	WB (1:1000)	Cell Signaling Technology	4129s
Arginase 1	WB (1:1000); IHC, IF (1:200)	Cell Signaling Technology	93668s
CSF1R	WB (1:1000)	Cell Signaling Technology	3152s
BrdU	IF (1:200)	BioRad	MCA2483GA
β-actin	WB (1:5000)	Santa Cruz	sc-47778
E-Cadherin	IF (1:200)	Cell Signaling Technology	3195s
E-Cadherin	IF (1:100)	Santa Cruz	sc-21791
Pan-CK	IF (1:200)	Abcam	ab9377
Pan-CK	IF (1:200)	BioLegend	914204
F4/80	IF (1:200)	BioRad	MCA497GA
F4/80-PE	FCM (1:100)	BioLegend	123110
CD206	IF (1:200)	BioRad	MCA2235GA
CD206-AlexaFluor488	FCM (1:100)	BioLegend	141710
HO-1	IF (1:200)	Enzo Life Sci	ADI-SPA-111
HRP-goat anti-mouse IgG	WB (1:10000)	Biolegend	405306
HRP-goat anti-rabbit IgG	WB (1:10000)	Cell Signaling Technology	7074s
Purified Mouse IgG1, Isotype Ctrl		BioLegend	401401
Purified Mouse IgG2a, Isotype Ctrl		BioLegend	401501
Purified Mouse IgG2b, Isotype Ctrl		BioLegend	400301
Purified rabbit polyclonal isotype Ctrl		BioLegend	910801
Purified Rat IgG1, Isotype Ctrl		BioLegend	400401
Purified Rat IgG2a, Isotype Ctrl		BioLegend	400501
Alexa Fluor® 594 Donkey anti-rabbit IgG	IF (1:300)	BioLegend	406418
Alexa Fluor® 488 Donkey anti-rabbit IgG	IF (1:300)	BioLegend	406416
Alexa Fluor® 594 goat anti- mouse IgG	IF (1:300)	BioLegend	405326
DyLight™ 488 goat anti- mouse IgG	IF (1:300)	BioLegend	405310
Alexa Fluor® 488 goat anti- rat IgG	IF (1:300)	BioLegend	405418
Alexa Fluor® 594 goat anti- rat IgG	IF (1:300)	BioLegend	405422

Supplementary Table 2. qRT-PCR primer sequences

Gene name		sequence
mCCL2	F	5'- TGT AGT TTT TGT CAC CAA GCT CA-3'
	R	5'-GTG CTT GAG GTG GTT GTG GA-3'
mCCL5	F	5'-CTT GCA GTC GTG TTT GTC ACT-3'
	R	5'-CCG AGT GGG AGT AGG GGA TT-3'
mCXCL9	F	5'-CAG CTC TGC CAT GAA GTC CG-3'
	R	5'-AGG GTT CCT CGA ACT CCA CAC-3'
mCXCL10	F	5'-TCA TCC TGC TGG GTC TGA GT-3'
	R	5'-CAT CGT GGC AAT GAT CTC AAC A-3'
mIL-10	F	5'-AGT GGA GCA GGT GAA GAG TGA TTT-3'
	R	5'-CTA TGC AGT TGA TGA AGA TGT C-3'
mIL-6	F	5'-ACA AAG CCA GAG TCC TTC AGA GA-3'
	R	5'-CTG TTA GGA GAG CAT TGG AAA TTG-3'
mNOS2	F	5'-CCC TCC AGT GTC TGG GAG CA-3'
	R	5'-TGC TTG TCA CCA CCA GCA GT-3'
mCD86	F	5'-TGT TTC CGT GGA GAC GCA AG-3'
	R	5'-TTG AGC CTT TGT AAA TGG GCA-3'
mCCL22	F	5'-AGG TCC CTA TGG TGC CAA TGT-3'
	R	5'- CGG CAG GAT TTT GAG GTC CA-3'
mTnfα	F	5'-CTT CTG TCT ACT GAA CTT CGG G-3'
	R	5'-CAG GCT TGT CAC TCG AAT TTT G-3'
mIL-1β	F	5'-ACC CCA AAA GAT GAA GGG CTG -3'
	R	5'-TAC TGC CTG CCT GAA GCT CT-3'
mArg1	F	5'-GGT TCT GGG AGG CCT ATC TT-3'
	R	5'-CAC CTC CTC TGC TGT CTT CC-3'
mRelmα	F	5'-CCA TAG AGA GAT TAT CGT GGA-3'
	R	5'-TGG TCG AGT CAA CGA GTA AG-3'
mCCR2	F	5'-TCC TTG GGA ATG AGT AAC TGT GT-3'
	R	5'-TGG AGA GAT ACC TTC GGA ACT T-3'
mTgfβ1	F	5'-GCT GAA CCA AGG AGA CGG AAT A-3'
	R	5'-GAG TTT GTT ATC TTT GCT GTC ACA AGA-3'
mFoxp3	F	5'-GGC CCT TCT CCA GGA CAG A-3'
	R	5'-GCT GAT CAT GGC TGG GTT GT-3'
mPDL1	F	5'-TGC GGA CTA CAA GCG AAT CAC G-3'
	R	5'-CTC AGC TTC TGG ATA ACC CTC G-3'
mPD1	F	5'-CGG TTT CAA GGC ATG GTC ATT GG-3'
	R	5'-TCA GAG TGT CGT CCT TGC TTC C-3'
mGAPDH	F	5'-TGA AGC AGG CAT CTG AGG G-3'
	R	5'-CGA AGG TGG AAG AGT GGG AG-3'

Supplemental File 1_Uncropped WB

Fig. 1H

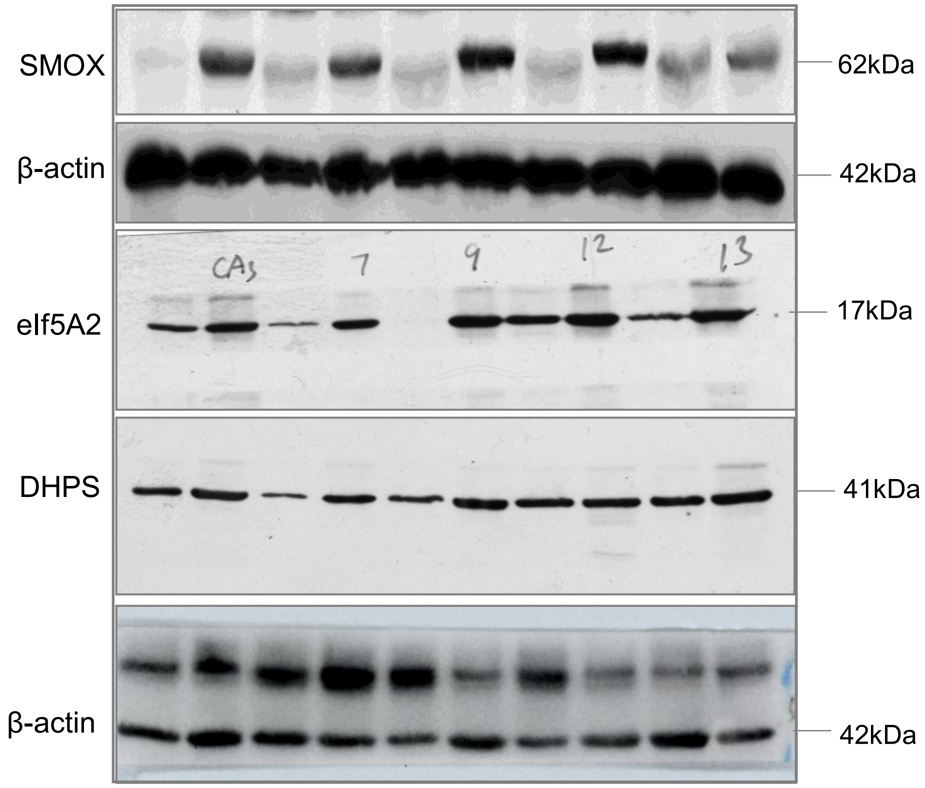

Fig. 3C

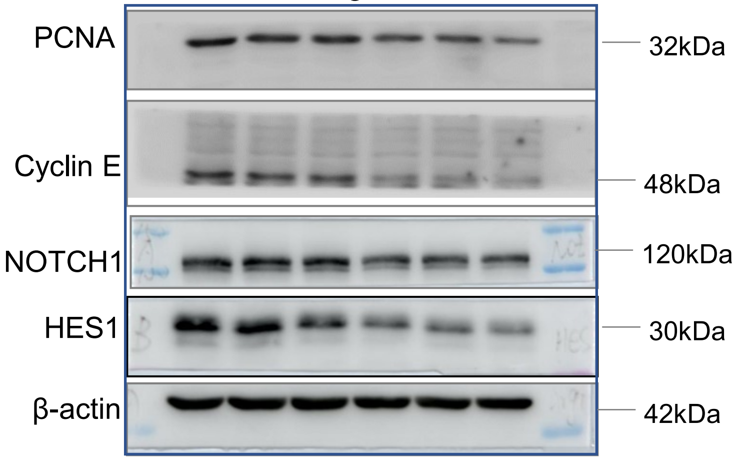

Fig. 3E

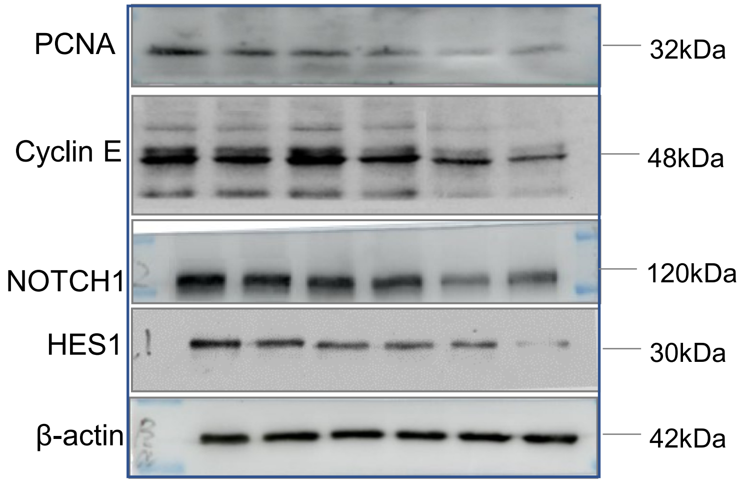

Fig. 3A

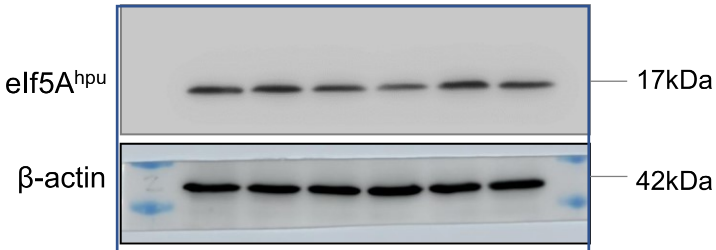

Fig. 3B

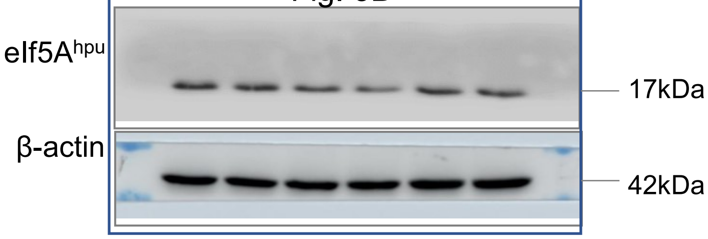

Fig. 4A

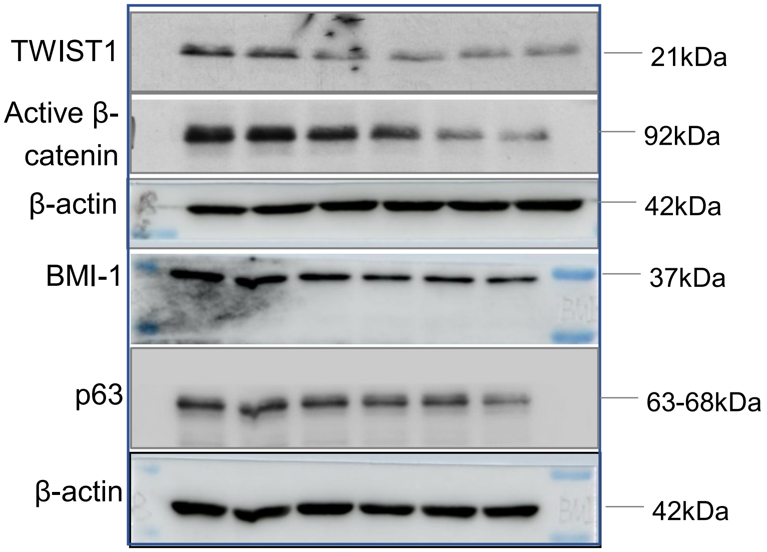

Fig. 4C

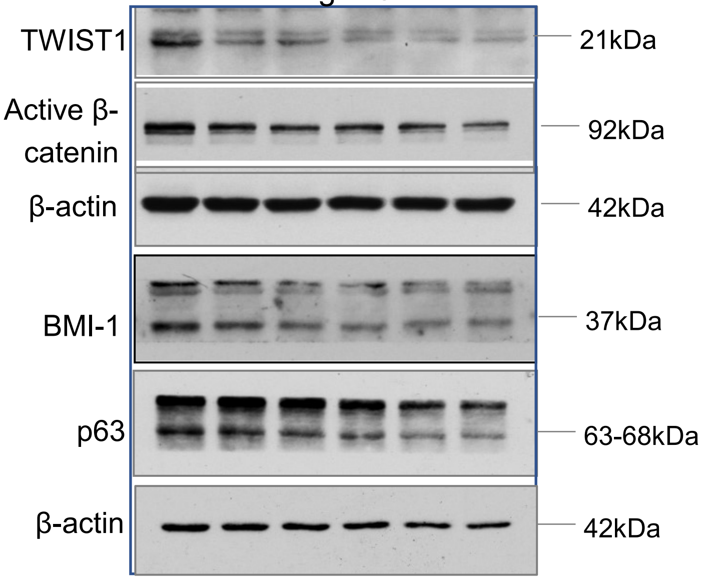

Fig.5E

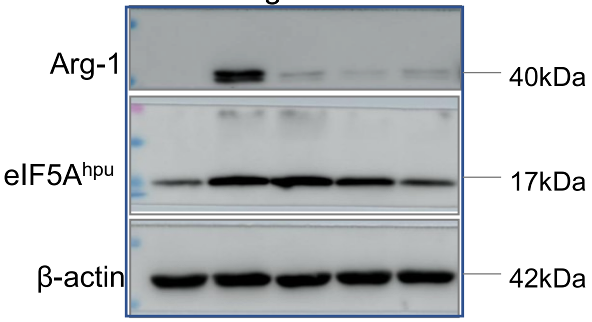

Fig. 5F

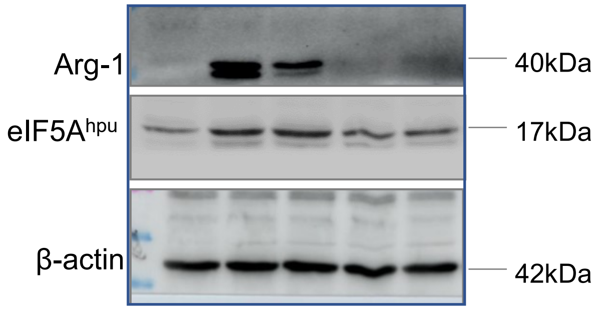

Fig. 5H

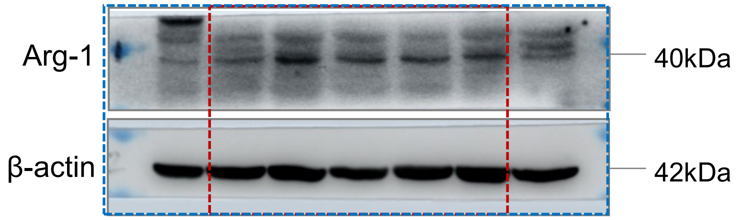

Fig. 5H

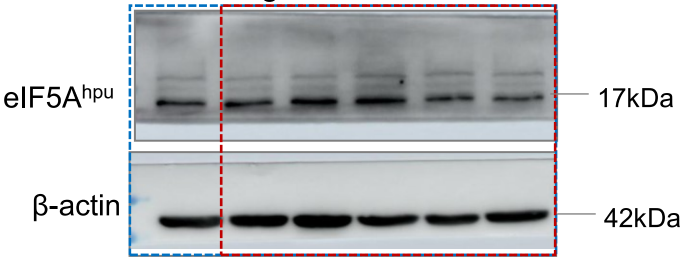

Supplementary Fig. 3

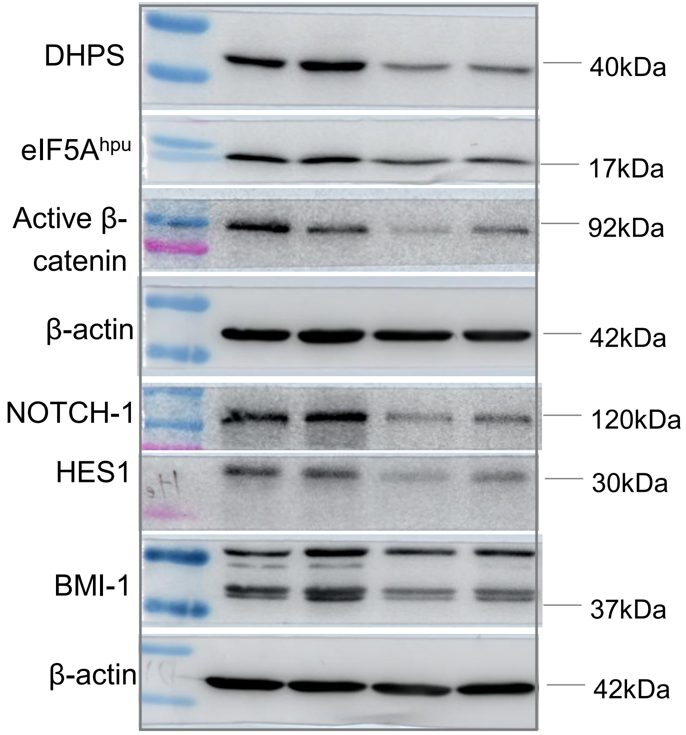

Supplementary Fig. 7

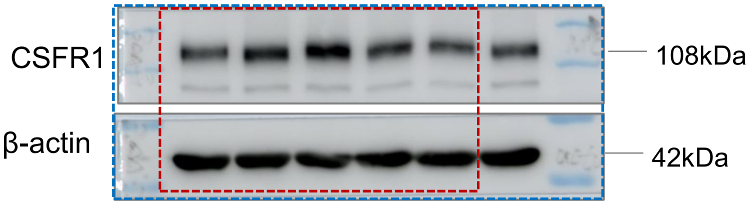

Supplement: Supplementary file 1 — Supplementary materials [file 41419_2023_6109_MOESM1_ESM.pdf]
